# Loss-of-function mutation in anthocyanidin reductase activates the anthocyanin synthesis pathway in strawberry

**DOI:** 10.1186/s43897-024-00106-2

**Published:** 2024-09-14

**Authors:** Pengbo Xu, Maobai Li, Chao Ma, Xinyu Li, Peng Bai, Anqi Lin, Chong Wang, Liqing Zhang, Huiyun Kuang, Hongli Lian

**Affiliations:** 1 Shanghai Collaborative Innovation Center of Agri-Seeds, School of Agriculture and Biology, Shanghai Jiao Tong University, Shanghai, China; 2 Shanghai Agricultural Technology Extension and Service Center, Shanghai, China; 3 Dandong Academy of Agricultural Sciences, Dandong, China; 4 Forestry and Fruit Tree Research Institute, Shanghai Academy of Agricultural Sciences, Shanghai, China; 5 Shanghai Agricultural Science and Technology Service Center, Shanghai, China

**Keywords:** Anthocyanins, Anthocyanidin reductase, Darker red fruit, MYB105, Pink fruit, Woodland strawberry

## Abstract

Fruit color substantially affects consumer preferences, with darker red strawberries being economically more valuable due to their higher anthocyanin content. However, the molecular basis for the dark red coloration remains unclear. Through screening of an ethyl methanesulfonate mutant library, we identified a *rg418* mutant, that demonstrated anthocyanin accumulation during early fruit development stages. Furthermore, the ripening fruits of this mutant had higher anthocyanin content than wild-type (WT) fruits. An analysis of flavonoid content in WT and *rg418* mutant fruits revealed substantial changes in metabolic fluxes, with the mutant exhibiting increased levels of anthocyanins and flavonols and decreased levels of proanthocyanidins. Bulked sergeant analysis sequencing indicated that the mutant gene was anthocyanidin reductase (*ANR*), a key gene in the proanthocyanidin synthesis pathway. Furthermore, transcriptome sequencing revealed the increased expression of *MYB105* during the early development stage of mutant fruits, which promoted the expression of *UFGT* (*UDP-glucose flavonoid 3-O-glucosyltransferase*), a key gene involved in anthocyanin synthesis, thus substantially enhancing the anthocyanin content in the mutant fruits. Additionally, mutating *ANR* in a white-fruited strawberry variant (*myb10* mutant) resulted in appealing pink-colored fruits, suggesting the diverse roles of *ANR* in fruit color regulation. Our study provides valuable theoretical insights for improving strawberry fruit color.

**Supplementary Information:**

The online version contains supplementary material available at 10.1186/s43897-024-00106-2.

## Core

The sensory quality of fruit, particularly its color, has become a significant focus in strawberry breeding. By mutating the anthocyanin reductase gene in strawberries that are naturally red or white in color, we observed darker red and pleasing pink fruits. This finding highlights that targeting this gene may be a promising approach for modifying the color profile of strawberry fruits.

## Genes & Accession Numbers

Information for the genes (*ANR, MYB105, UFGT, MYB10,* and *bHLH3*) discussed in this article is available in the Genomic Database for Strawberries (GDS, http://eplant.njau.edu.cn/strawberry/) under the following accession numbers: *ANR* (FvesChr3G00071690), *MYB105* (FvesChr7G00341810), *UFGT* (FvesChr4G00131790), *MYB10* (FvesChr1G00306600), and *bHLH3* (FvesChr2G00208740).

## Introduction

The octoploid cultivated strawberry (*Fragaria × ananassa*), a member of the Rosaceae family, is a widely popular fruit among consumers. Strawberries are rich in various bioactive compounds, such as vitamin C, flavonols, proanthocyanidins, and anthocyanins. Anthocyanins, which are soluble in water, impart vibrant colors to the flowers, fruits, and leaves of the plant. These compounds not only improve the aesthetic appeal of plants, attracting insects, birds, and other animals for pollination and seed dispersal, but also protect the plants from photooxidative damage caused by intense light exposure (Steyn et al. 2002; Giampieri et al. 2012). Furthermore, the consumption of fruits rich in anthocyanins can prevent chronic diseases, such as cancer and cardiovascular diseases (van Gils et al. 2005; Bertoia et al. 2016). Thus, the regulatory mechanism of anthocyanin accumulation remains a prominent area of research.

The synthesis of anthocyanins is a complex biochemical process that primarily begins with two organic substances: malonyl coenzyme A derived from the fatty acid metabolic pathway and coumaroyl coenzyme A obtained from the phenylpropanoid pathway. The phenylpropanoid pathway is a critical secondary metabolic pathway in plants, and it is essential for the synthesis of various compounds, including anthocyanins and proanthocyanidins. During the synthesis of anthocyanins and proanthocyanidins, malonyl coenzyme A and coumaroyl coenzyme A undergo a series of enzymatic reactions catalyzed by chalcone synthase (CHS), chalcone isomerase (CHI), flavanone-3-hydroxylase (F3H), dihydroflavonol-4-reductase (DFR), and anthocyanidin synthase (ANS). These enzymatic reactions result in the formation of unstable anthocyanidins. The subsequent modification of unstable anthocyanidins can result in either the production of stable anthocyanins through the action of UDP-glucose flavonoid 3-O-glucosyltransferase (UFGT) or their condensation into proanthocyanidins facilitated by anthocyanidin reductase (ANR) (Springob et al. 2003; Fukusaki et al. 2004; Schaart et al. 2013; Zhang et al. 2014).

The synthesis of anthocyanins and proanthocyanidins is regulated by the expression of a set of specific enzyme genes and influenced by diverse factors such as hormones, environmental conditions, and transcription factors (Yang et al. 2022). A key regulator of anthocyanin and proanthocyanidin synthesis is the MBW (MYB-bHLH-WD40) complex, composed of MYB, bHLH, and WD40 proteins (Sunil and Shetty 2022). In *Arabidopsis*, any of the three bHLH transcription factors—bHLH3, EGL3, or GL3—can combine with MYB75 and the WD40 protein TTG1 to form this MBW complex, which then promotes anthocyanin synthesis (Gonzalez et al. 2008). In grapevine, the transcription factor VvMYC1 interacts with several MYB proteins—VvMYB5a, VvMYB5b, VvMYBA1/A2, and VvMYBPA1—to activate the expression of genes essential for the synthesis of anthocyanins and proanthocyanidins (Hichri et al. 2010). In apple, light-induced expression of *MdMYB1* enhances anthocyanin accumulation in the fruit’s skin (Takos et al. 2006). In strawberry, FaMYB1, the first identified MYB transcription factor, inhibits anthocyanin production (Aharoni et al. 2001). The expression of *FcMYB1*—an ortholog of *FaMYB1*—is higher in *Fragaria chiloensis* than in the red strawberry *Fragaria × ananassa* cv. Camarosa, indicating its role in the fruit’s white coloration (Salvatierra et al. 2013). Another MYB transcription factor, termed FaMYB10 in octoploid cultivated strawberries and FvMYB10 in woodland strawberries, has been shown to enhance anthocyanin accumulation (Lin et al. 2010; Lin et al. 2014). Overexpression of FvMYB10 results in color changes in various parts of strawberry plants such as petioles, leaves, pistil stigmas, petals, and fruit flesh (Lin et al. 2014).

Sequence analysis of *FvMYB10* in various diploid white-fruited strawberry strains has revealed four types of sequence variations. The first type is a mutation within the conserved domain of the R2R3-MYB transcription factor, specifically a guanine to cytosine mutation at position 35 in the coding sequence (CDS) region, which affects a tryptophan residue (Hawkins et al. 2016). The second type is the insertion of an LTR-type transposon within the third exon. The third type is the insertion of an adenine base at position 329 in the CDS region. The fourth type is the deletion of a large fragment, which results in the loss of *FvMYB10* function (Castillejo et al. 2020). Similarly, an eight-base insertion in the sequence of *FaMYB10* found in the octoploid white strawberry variety ‘Snow Princess’ leads to premature translation termination, resulting in white fruit coloration (Wang et al. 2020). These variations in the CDS region can impair the functionality of crucial R2R3-MYB transcription factors, leading to color variations. Moreover, variations in the promoter region have been shown to affect the expression levels of *R2R3-MYB* genes, influencing color variations across different species (Kobayashi et al. 2004; Butelli et al. 2012; Jung et al. 2019; Xu et al. 2019; Guo et al. 2020). Specifically, in the *Fragaria × ananassa* cv. Camarosa variety, the promoter regions of *FaMYB10-2*, a key homolog of *FvMYB10*, contain a 23-kb transposon sequence with hormone- and sugar-response elements. This configuration results in the high expression of *FaMYB10-2*, promoting anthocyanin synthesis in both the skin and flesh of the fruits (Castillejo et al. 2020).

Mutations in *FvMYB10* affect anthocyanin accumulation in fruits but not in the petioles (Xu et al. 2014; Hawkins et al. 2016). This distinction led to the identification of another R2R3-MYB transcription factor, FvMYB10L, which is specifically expressed in petioles and regulates their anthocyanin content. Mutations in the gene *FvMYB10L* result in sustained greening of the petioles (Luo et al. 2023). Furthermore, the expression levels of *MYB10*, *CHS*, *DFR*, and *UFGT* in fruits have been linked to either transient overexpression or silencing of *FvMYB79*, affecting anthocyanin synthesis (Cai et al. 2021). In octoploid strawberries, the protein FaMYB63 directly activates the promoter of *FaMYB10*, highlighting its role in the regulation of anthocyanin accumulation (Wang et al. 2022a, b).

In addition to MYB transcription factors, three bHLH transcription factors—FvbHLH3, FvbHLH33, and FvMYC1—have been identified in strawberries as potential players in flavonoid synthesis and metabolism. These factors are known to interact with various MYB transcription factors to form complexes (Lin et al. 2014; Xu et al. 2021). Notably, FvbHLH33 significantly enhances the promoter activities of *DFR1* and *UFGT* when co-expressed with FvMYB10 (Lin et al. 2014). Our previous study reported that when FvbHLH33 is co-expressed with FvMYB22, FvMYB64, or FvMYB105, it not only boosts the promoter activity of the *DFR2* gene but also enhances proanthocyanidin accumulation in fruits (Xu et al. 2021). However, strawberry fruits from *FvbHLH33-RNAi* transgenic lines did not display significant color differences compared with the control, suggesting functional redundancy with other factors (Lin et al. 2014). Schaart et al. observed that FabHLH3 can interact with FaMYB9, FaMYB11, and FaMYB5 to regulate proanthocyanidin synthesis in immature fruits (Schaart et al. 2013). Recently, Yue *et al.* discovered that the transient overexpression of *FaMYB9*, *FaMYB11*, and *FaMYB5* promotes the synthesis of both proanthocyanidins and anthocyanins in fruits (Yue et al. 2023). In addition, FabHLH3 can interact with FaMYB123, activating the expression of *FaMT1*, which plays a crucial role in the later stages of the flavonoid synthesis pathway (Martínez et al. 2023).

In addition to altering the expression of *MYB* genes or the function of MYB proteins, direct modifications to enzyme genes involved in the anthocyanin synthesis pathway can affect anthocyanin levels in strawberry fruit. Recently, we identified a mutation in the flavanone-3-hydroxylase (F3H) protein at position 130, where an arginine residue is replaced by a histidine residue. This mutation reduces the catalytic activity of F3H, disrupting the anthocyanin synthesis pathway and imparting a pinkish hue to the fruits (Xu et al. 2023). Furthermore, the glutathione transferase gene *RAP* plays a crucial role in transporting anthocyanins to vesicles for storage. Luo *et al.* found that mutations in the *RAP* gene in strawberry plants prevented anthocyanin accumulation in the petioles, causing the fruit to change from red to white (Luo et al. 2018). Using CRISPR–Cas9 technology to knock out *RAP* and its homologous genes in octoploid strawberries resulted in the creation of white-fruited strawberry germplasm (Gao et al. 2020). During the development and ripening of strawberry fruits, proanthocyanidins primarily accumulate in the early stages, whereas anthocyanins accumulate during the later turning and ripening stages (Xu et al. 2021). Moreover, the overexpression of *RAP* led to excessive anthocyanin accumulation in both the skin and flesh of the fruit in these early stages (Gao et al. 2020). However, FvMYB10 was not involved in anthocyanin accumulation at this stage, suggesting the role of other potential factors in this process.

In this study, we screened an ethyl methanesulfonate (EMS) mutant library and identified the *rg418* mutant, notable for its red stigmas of pistils during flowering and early-stage anthocyanin accumulation in fruits. Bulked-segregant analysis sequencing (BSA-Seq) identified the causal gene as *ANR*, whereas transcriptome sequencing showed a significant increase in *MYB105* expression in the *rg418* (*anr*) mutant. MYB105 specifically binds to the promoter of *UFGT*, enhancing its expression and thus promoting anthocyanin synthesis when the pathway for proanthocyanidin synthesis is inhibited. In addition, compared with WT RG (Ruegen), the *anr* mutant fruits exhibited a darker red hue during the ripening stage. By contrast, fruits with mutations in both *ANR* and *MYB10* displayed a pink coloration. Our findings provide valuable insights for the molecular breeding of strawberry germplasm in various colors.

## Results

### Identification and phenotypic analysis of the red flesh mutant

**Fig. 1:**
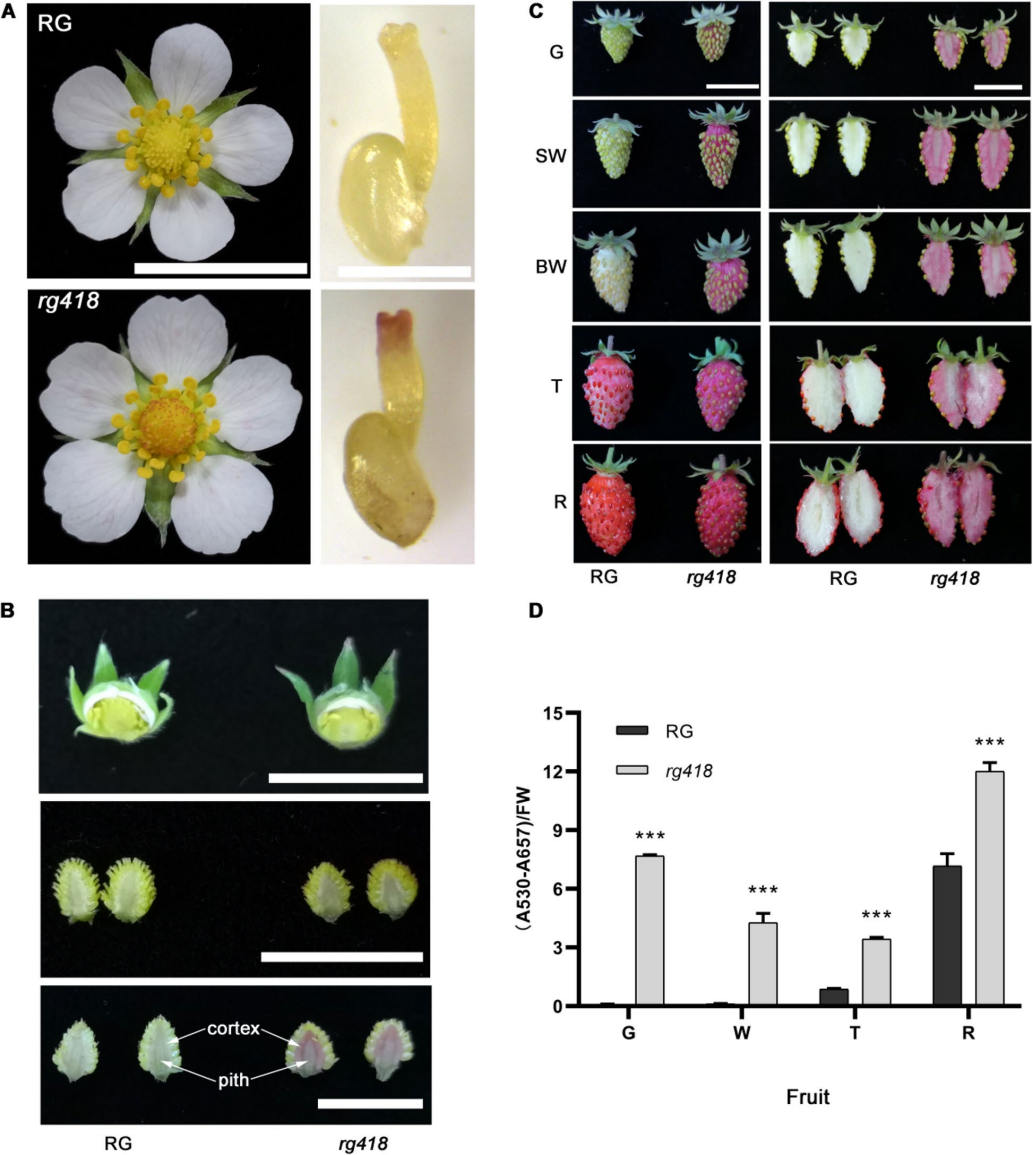
The phenotypic analysis of *rg418* mutant and wild-type RG. **A** The phenotype of flower and pistil stigma. **B** The receptacle phenotype in early development stages. **C** The fruits phenotype at different developmental stages. **D** The content of anthocyanins in fruits at different developmental stages. G: green stage, SW: small white stage, BW: Big white stage; T: turning stage, and R: ripening stage. FW: Fresh Weigh. Data in (**D**) was mean ± SD, *n* = 3. ***, *p* <0.001, Student’s t-test. Bars in (**A**-**C**) are 1 cm. RG: Ruegen. Strawberry fruits are formed by the development and expansion of the receptacle. The outer of receptacle was cortex; and the inner of receptacle was pith

We conducted EMS mutagenesis using the woodland strawberry RG as the WT to explore genetic factors influencing fruit development and quality. In the M2 mutant populations, we identified a mutant with a unique phenotype of anthocyanin accumulation in the pistil’s stigma during flowering, contrasting with the RG flowers that showed no anthocyanin accumulation (Fig. 1A). This mutant, originating from RG with an initial number of 418, was named the *rg418* mutant. To further investigate anthocyanin accumulation, we dissected unopened flower buds and observed no anthocyanin in the stigmas and receptacles of both the *rg418* mutant and RG (Fig. 1B, upper panel). Similarly, anthocyanins were found to be lacking in the receptacles upon opening the flowers on the blooming day (Fig. 1B, middle panel). However, by the third day post-blooming, the accumulation of anthocyanin was evident in both the cortex and pith regions of the receptacles in the *rg418* mutant, whereas the RG receptacle remained devoid of anthocyanin (Fig. 1B, lower panel). These observations indicated that anthocyanins begin to accumulate early in the fruit development of the mutant.

To thoroughly examine the fruit color phenotype of the *rg418* mutant, we tracked its development from the green to the ripening stages. Throughout this period, the skin and flesh of the *rg418* mutant consistently exhibited a red phenotype. By contrast, RG fruits did not accumulate anthocyanins during the transition from green to white stages but started to show anthocyanin accumulation in the skin during the turning and ripening stages. Notably, the flesh of RG fruits did not accumulate anthocyanins at any stage of development (Fig. 1B and C). Quantitative analysis revealed that the total anthocyanin content in RG fruits peaked at the ripening stage, whereas the *rg418* mutant already displayed anthocyanin levels at the green stage comparable to those noted in RG at maturity. Furthermore, in the *rg418* mutant, anthocyanin content decreased during the white and turning stages relative to the green stage. However, by the maturation stage, anthocyanin accumulation in the *rg418* mutant was approximately twice that of the green stage (Fig. 1D). These findings suggested that anthocyanins, products of the flavonoid metabolic pathway, accumulate abnormally in the *rg418* mutant, indicating a potential disruption in this metabolic pathway.

### Flavonoid metabolic flow is substantially altered in *rg418* mutants

Considering the notably darker red color observed in the ripening *rg418* mutant fruits compared with RG fruits (Fig. 1D), we measured the anthocyanin composition and content. Our analysis identified cyanidin, pelargonidin, and peonidin as the primary pigments in both RG and *rg418* mutant fruits. In RG, the ratio of these pigments was approximately 4:4:1. In the *rg418* mutant, this ratio shifted to 3:6:1, highlighting a difference in anthocyanin pigment proportions between the two. Moreover, the *rg418* mutant showed increased levels of these pigments compared with RG: cyanidin content was 1.56 times higher, pelargonidin content was 2.97 times higher, and peonidin content was 2.17 times higher (Table 1). Overall, the total anthocyanin content was 538.94 μg/g in RG fruits, whereas it reached 1213.67 μg/g in the *rg418* mutant fruits, marking a 2.25-fold increase (Table 1).

**Table 1:** The content of each pigment component in strawberry petioles and fruits

Anthocyanin composition	Petiole		Fruit	
[ug/g FW]	RG	*rg418*	RG	*rg418*
Cya-3,5-O-diglu	92.32±13.346	162.72±5.874 **	0.40±0.0123	1.45±0.057 ***
Cya-3-O-glu	89.66±10.382	114.77±4.673 *	124.64±6.778	135.29±3.851 ns
Cya-3-O-(6-O-malonyl)-glu	0.16±0.037	0.35±0.112 *	110.82±11.929	230.17±12.800 ***
**Total Cyanidin**	**182.14±22.916**	**277.85±3.451** **	**235.85±16.681**	**366.91±16.081** ***
Pel-3-O-glu	5.74±0.835	13.01±1.587 **	77.02±3.070	88.97±2.005 **
Pel-3,5-O-diglu	0.56±0.135	1.21±0.142 **	1.22±0.033	10.61±0.250 ***
Pel-3-O-(6-O-malonyl)-glu	0.03±0.006	0.07±0.017 *	168.57±31.066	633.25±36.759 ***
**Total Pelargonidin**	**6.33±0.834**	**14.29±1.492** **	**246.80±33.973**	**732.82±34.980** ***
Peo-3,5-O-diglu	190.15±8.537	204.88±3.127 *	0.34±0.022	2.68±0.038 ***
Peo-3-O-gal	7.54±1.30	13.31±1.476 **	0.31±0.024	1.32±0.091 ***
Peo-3-O-glu	47.00±2.186	53.68±2.958 *	38.56±0.665	45.69±1.094 ***
Peo-3-O-(6-O-malonyl)-glu	1.57±0.132	4.06±0.759 **	18.30±2.231	74.87±2.825 ***
**Total Peonidin**	**246.26±7.045**	**275.93±6.560** **	**57.50±2.913**	**124.55±2.086** ***
**Total anthocyanin**	**434.18±62.277**	**566.85±76.551** **	**538.94±71.748**	**1213.67±207.082** ***
Quercetin-glu	0.89±0.301	45.14±18.214 ***	0.32±0.175	123.69±24.611 ***
Procyanidin B3	153.66±54.707	64.36±38.359 ***	2.96±1.893	1.19±1.193 ns
Procyanidin B1	175.50±48.261	27.42±13.520 ***	1.97±2.190	0.36±0.072 ***

*Cya-3,5-O-diglu* a cyanidin-3,5-O-diglucoside, *Cya-3-O-glu* cyanidin-3-O-glucoside, *Cya-3-O-(6''-O-malonyl)-glu* cyanidin-3-O-(6''-O-malonyl)-glucoside, *Pel-3-O-glu* pelargonidin-3-O-glucoside, *Pel-3,5-O-diglu* pelargonidin-3,5-O-diglucoside, *Pel-3-O-(6-O-malonyl)-glu* pelargonidin-3-O-(6''-O-malonyl)-glucoside, *Peo-3,5-O-diglu* peonidin-3,5-O-diglucoside, *Peo-3-O-glu* peonidin-3-O-glucoside, *Peo-3-O-gal* peonidin-3-O-galactosidase, *Peo-3-O-(6-O-malonyl)-glu* peonidin-3-O-(6''-O-malonyl)-glucoside, *Quercetin-glu* quercetin-glucoside. Student's t test was used for pairwise comparisons between RG and *rg418*.  *, *p*<0.05; **, *p*<0.01; ***, *p*<0.001; ns, no significant. RG: Ruegen.

Anthocyanins also accumulate in the petioles of RG. Visual observations revealed that the petiole color of both RG and the *rg418* mutant appeared almost identical, with no noticeable differences (Supplemental Fig. S1). However, upon analyzing the anthocyanin components in the petioles, we found that cyanidin and peonidin were the primary pigments, with pelargonidin present in very low quantities (Table 1). In the *rg418* mutant, the total peonidin content in the petioles was slightly higher, showing an increase of 1.12 times compared with RG, whereas the total cyanidin content exhibited a more substantial increase of 1.53 times. Overall, the total anthocyanin content in RG petioles was 434.18 μg/g, whereas in the *rg418* mutant, it reached 566.85 μg/g, representing a 1.31-fold increase compared with RG (Table 1).

We also analyzed the content of quercetin and procyanidins in both petioles and fruits. In RG, the quercetin levels were low in both petioles and ripe fruits, measuring less than 1 μg/g. However, the *rg418* mutant showed a substantial increase in quercetin content, reaching 45.14 μg/g in petioles and 123.69 μg/g in fruits, representing a 50-fold and 386-fold increase, respectively (Table 1). Additionally, the analysis of procyanidin content revealed that the levels of procyanidin B1 and B3 were below 3 μg/g in the ripening fruits and were even lower in the *rg418* mutant than that in RG. Procyanidins were more concentrated in the petioles than in fruits. In the *rg418* mutant petioles, the levels of procyanidin B1 and B3 were significantly reduced by 6.40-fold and 2.39-fold, respectively, compared with RG petioles (Table 1). Flavonols, such as quercetin, are also products of the flavonoid metabolic pathway. Although quercetin levels were typically below 1 μg/g in RG, its levels in the fruits and petioles of the *rg418* mutant significantly increased to 123.69 μg/g and 45.14 μg/g, respectively. This variation in the contents of quercetin, procyanidins, and anthocyanidins suggests significant alterations in the flavonoid metabolic pathway in the *rg418* mutant compared with RG, indicating a redirected metabolic flow within this pathway.

### Identification of causal mutation in the *rg418* mutant

To identify and isolate the gene responsible for anthocyanin accumulation in the *rg418* mutant, we crossed the mutant with WT RG. The resulting F1 generation displayed the same phenotype as RG. However, in the F2 generation, which consisted of 280 plants, clear phenotypic segregation was observed: 202 plants exhibited the WT RG phenotype and 78 plants showed the *rg418* mutant phenotype. The observed segregation ratio of 2.59:1 (202:78) is close to the expected 3:1 ratio (*X*^2^ = 0.27 < *X*^2^_(0.05,1)_ = 3.84), indicating that *rg418* mutation is a monogenic recessive trait. For further analysis, DNA was extracted from 45 F2 mutant plants, pooled in equal amounts, and analyzed using the MutMap method (Abe et al. 2012; Tribhuvan et al. 2018). DNA from WT RG served as the reference in the WT pool. The sequencing data from both pools were analyzed using the MutMap pipeline (Sugihara et al. 2022), which identified candidate mutant genes within a specific 2.02-Mb region on chromosome 3 (Fvb3: from “1276157” to “3348108”; Supplemental Fig. S2).

**Fig. 2:**
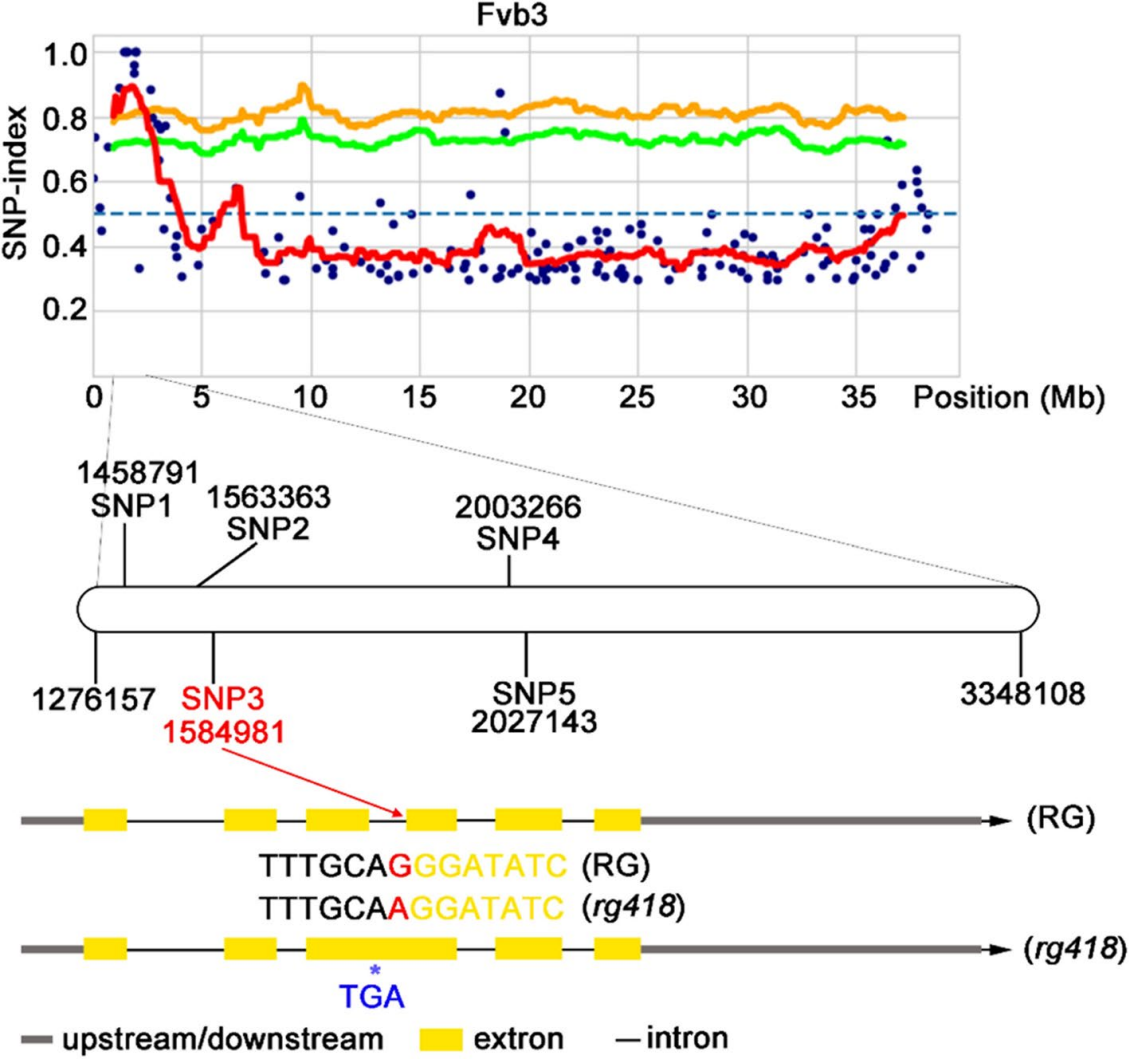
The mapping of mutant gene of *rg418* mutant. The mutagenic gene is located in 1276157 bp to 3348108 bp interval of chromosome 3, in which a total of five SNPs have an index equal to 1. The SNP3 is located exactly at the splice site of the third intron of *ANR* gene. The G to A mutation causes the third intron to be preserved and not cut. The red line represents the mean of SNP-index. The orange line and green line represent the mean p-value of SNP was 0.01 and 0.05, respectively. The black sequence of TTTGCA represents the third intron sequence, and the yellow sequence of GGATATC represents the fourth exon sequence. SNP: Single Nucleotide Polymorphism. TGA: termination codon. RG: Ruegen

Within the identified candidate interval, we observed five mutant loci, each with an SNP (single nucleotide polymorphism) index of 1. Of these, three SNPs were located in the intergenic region, not affecting the coding sequences. The remaining two SNPs, SNP3 and SNP4, were of particular interest. SNP4 was located within the coding region of the gene *FvH4_3g03590* and resulted in nonsynonymous mutations altering amino acids. This gene is functionally annotated as an S-receptor-like serine/threonine-protein kinase. SNP3 was located at the splice site of the third intron in the anthocyanidin reductase (*ANR*, FvH4_3g02980) gene (Fig. 2 and Supplemental Table S1). A mutation at this site could cause errors in intron splicing. To explore this possibility, we extracted total RNA from the fruits of both RG and the *rg418* mutant, converted it to cDNA, and then amplified and sequenced the *ANR* gene from each line. The sequencing results indicated that the *rg418* mutant’s *ANR* gene retained the third intron within its CDS sequence, unlike the RG’s *ANR* sequence (Supplemental Fig. S3). Translation of this mutant *ANR* sequence led to premature termination due to a frame shift mutation caused by intron retention (Supplemental Fig. S4). Given the crucial role of *ANR* in the proanthocyanidin synthesis pathway and the observed significant reduction in proanthocyanidin content in the *rg418* mutant (Table 1), it is plausible that *ANR* is the mutagenic gene in the *rg418* mutant (henceforth referred to as the *anr* mutant).

### Anthocyanin pathway is active in the *anr* mutant

**Fig. 3:**
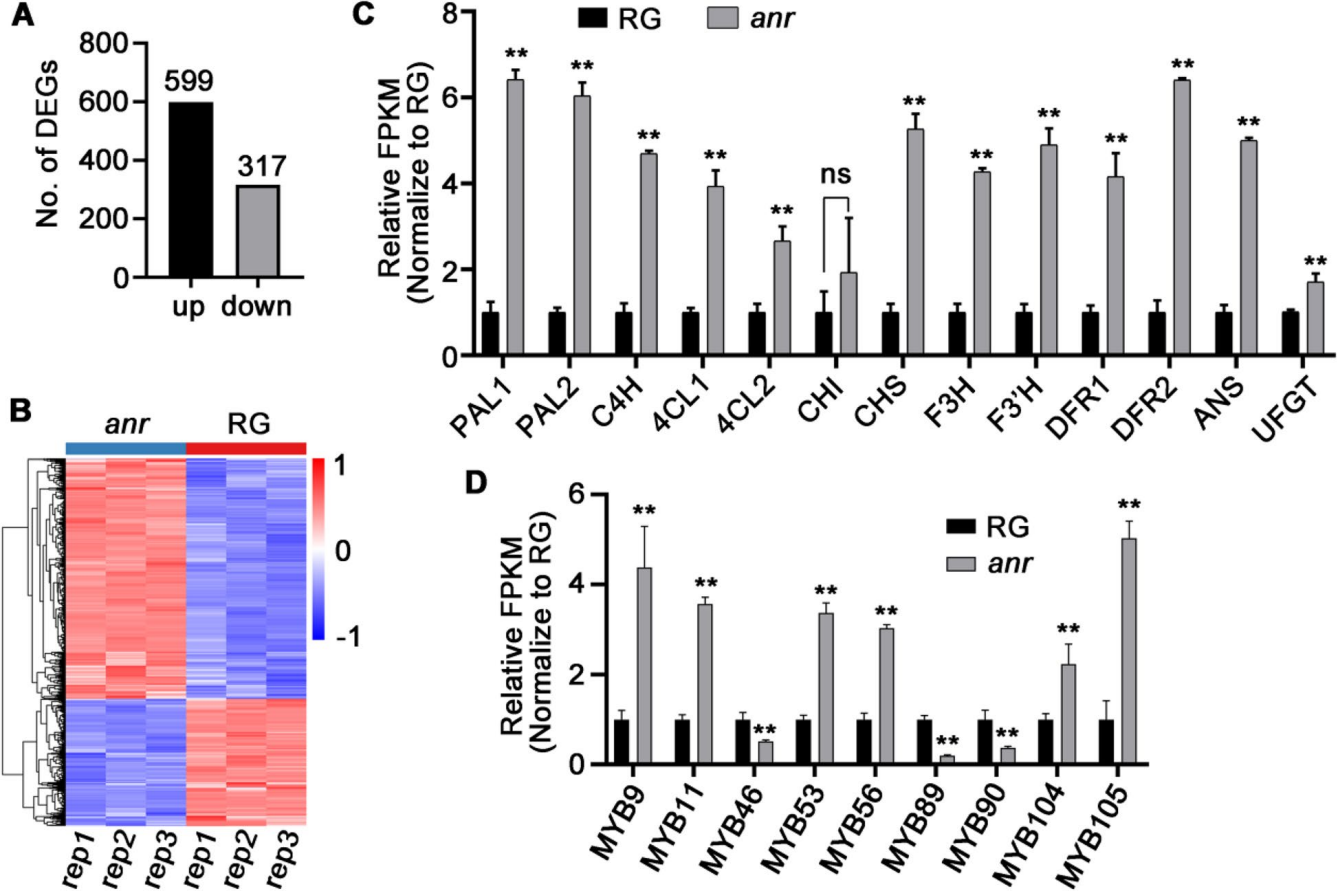
The anthocyanin pathway is activated in the *anr* mutant. **A** The number of differential expression genes (DEGs) between RG and *anr* mutant. up: up-regulated; down: down-regulated. **B** The heatmap of DEGs. rep1: repeat 1; rep2: repeat 2; rep3: repeat 3. **C** The enzyme genes related to anthocyanin synthesis were expressed at higher levels in the *anr* mutant than in RG. **D** The nine differentially expressed MYB transcription factors between RG and *anr* mutant. The vertical axis in (**C**) and (**D**) indicate the value of the FPKM in *anr* mutant relative to RG. FPKM: Fragments per Kilobase Million. The FPKM of genes in RG was normalized to 1. Data in (**C**) and (**D**) are mean ± SD, *n* = 3. **, *p* <0.01; ns, no significant; Student’s t-test. RG: Ruegen

The *anr* mutant fruits displayed significant anthocyanin accumulation during early development stages, unlike the WT RG fruits where such accumulation was absent. To explore the underlying molecular mechanisms, we conducted transcriptome sequencing on the receptacles of both RG and *anr* mutant fruits 3–5 days after flowering. The sequencing data were filtered and aligned to the woodland strawberry (*Fragaria vesca*) reference genome V6.0, achieving a mapping rate of over 98% for each library (Supplemental Table S2). Analysis of the transcriptome data revealed 916 differentially expressed genes (DEGs) between the two samples: 599 were upregulated and 317 were downregulated in the *anr* mutant compared with RG (Fig. 3A and Supplemental Table S3). The expression trends of these DEGs were consistent across three biological replicates (Fig. 3B), confirming the reliability of our data. KEGG pathway analysis indicated significant enrichment in pathways related to phenylalanine metabolism and flavonoid synthesis (Supplemental Fig. S5). Further investigation into the expression of structural genes involved in anthocyanin synthesis showed that with the exception of *CHI*, the expression levels of 12 genes from *PAL1* (*Phenylalanine ammonia lyase 1*) to *UFGT* were significantly higher in the *anr* mutant than in RG (Fig. 3C and Supplemental Table S4). To verify these findings, we performed RT-qPCR (reverse transcription-quantitative polymerase chain reaction) assays on nine randomly selected structural genes, confirming their differential expression (Supplemental Fig. S6). Additionally, we compared the expression of these structural genes during the ripening stages between RG and the *anr* mutant. At this later stage, the expression levels were even higher and notably greater in the *anr* mutant than in RG (Supplemental Fig. S6), suggesting enhanced activation of the anthocyanin synthesis pathway. Moreover, the fragments per kilobase per million (FPKM) value of *MYB10*, a critical gene in fruit anthocyanin synthesis, was very low in the early-stage transcriptome data (Supplemental Table S4), implying minimal influence at this stage. However, RT-qPCR showed increased expression of *MYB10* during ripening compared with early stages; these findings are consistent with those of previous research (Chen et al. 2020; Yue et al. 2023), highlighting the role of MYB10 in promoting anthocyanin accumulation during later stages. *MYB10* expression in the ripening fruits of the *anr* mutant was significantly higher than that in RG fruits (Supplemental Fig. S6), potentially explaining the elevated expression of genes associated with anthocyanin synthesis in the *anr* mutant. However, the expression level of *MYB10* was lower in the fruits in early development stages of the *anr* mutant and did not differ from that in RG fruits (Supplemental Table S4 and Supplemental Fig. S6), indicating that other factors might regulate anthocyanin accumulation at this stage.

### MYB105 promotes anthocyanin accumulation in the *anr* mutant during early stages of fruit development

In the flavonoid metabolic pathway, structural genes, such as *CHS*, *F3H*, *DFR*, *ANS*, and *UFGT*, are typically regulated by R2R3-MYB transcription factors. To explore which R2R3-MYB factors are involved in anthocyanin accumulation during the early development stages of the *anr* mutant fruit, we analyzed the DEGs and identified a total of nine R2R3-MYB transcription factors (Supplemental Table S5). Of these, three were downregulated and six were upregulated in *anr* mutant fruits (Fig. 3D). Notably, among the upregulated MYBs, three factors—MYB9 (FvesChr2G00217260.1), MYB11 (FvesChr6G00044270.1), and MYB105 (FvesChr7G00341810.1)—had previously been identified as candidates potentially involved in flavonoid metabolism (Xu et al. 2021). Thus, we decided to focus our subsequent research on these three transcription factors.

**Fig. 4:**
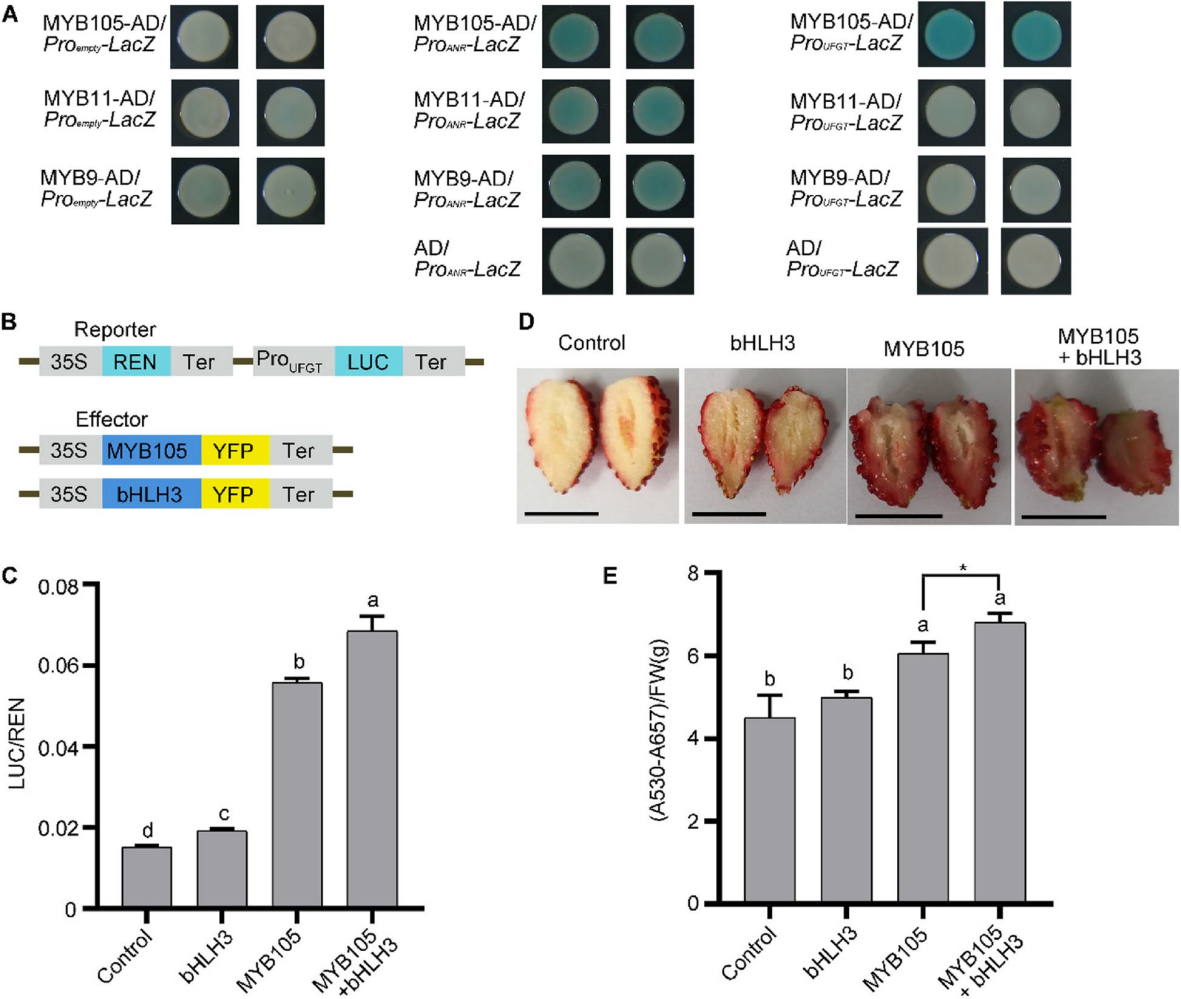
MYB105 activates *UFGT* and promotes anthocyanin accumulation. **A** Yeast one-hybrid showed that MYB9/11/105 could all bind the promoter of *ANR*, but only MYB105 could bind the promoter of *UFGT*. **B** Schematic illustration of reporter and effectors used in the dual luciferase experiment. REN: reilla luciferase; LUC: firefly luciferase. 35S-driven REN was used as an internal control. 35S: the cauliflower mosaic virus 35S promoter; Ter: terminator. **C** MYB105 activates the promoter of *UFGT*. The vertical axis indicates the ratio of firefly luciferase enzyme activity to renilla luciferase enzyme activity. **D** Transient injection experiments in fruit showed that MYB105 promoted anthocyanin accumulation. **E** The anthocyanin content of the fruits indicated in (**C**). Bar in (**D**) was 1 cm. Data in (**C**) and (**E**) are mean ± SD from three replicates. Different letters indicate significant differences between different groups (one-way ANOVA, Tukey test, *p* < 0.05). *, *p* < 0.05, Student's t-test. FW: Fresh Weigh

Proanthocyanidins and anthocyanins share many enzymes and substrates throughout their synthesis processes, differing only in the final reaction step. Specifically, ANR catalyzes the conversion of unstable anthocyanidins into proanthocyanidins, whereas UFGT glycosylates these unstable anthocyanidins to form stable anthocyanins (Schaart et al. 2013). To explore this further, we conducted a yeast one-hybrid assay to determine if three MYB transcription factors bind to the promoters of *UFGT* or *ANR*. The results indicated that MYB105 strongly binds to the *UFGT* promoter, whereas all three MYB transcription factors—MYB9, MYB11, and MYB105—could bind to the *ANR* promoter (Fig. 4A). Additionally, a quantitative analysis of the enzymatic activity of the reporter gene *LacZ* supported these findings (Supplemental Fig. S7). Moreover, MYB105 was shown to activate the expression of *LUC (luciferase)*, which is driven by the *UFGT* promoter (Fig. 4B and C). Similarly, MYB9, MYB11, and MYB105 all enhanced the expression of *LUC* driven by the *ANR* promoter, with this activation further increased in the presence of the bHLH transcription factor bHLH3 (Supplemental Fig. S8).

To further explore the role of MYB105 in enhancing anthocyanin accumulation, we performed a transient injection experiment on RG fruits. Given that RG fruits typically have red skin but their flesh lacks anthocyanin accumulation and appears white, we specifically focused on the flesh phenotype of the transiently injected fruits. Our observations showed that control fruits injected with YFP and bHLH3 did not exhibit significant anthocyanin accumulation in the flesh. By contrast, fruits injected with MYB105 alone or in combination with bHLH3 displayed noticeable anthocyanin accumulation in the flesh (Fig. 4D). We quantified the anthocyanin content in these fruits, finding that MYB105-injected fruits had higher anthocyanin levels than the control, and fruits injected with both MYB105 and bHLH3 had accumulated even greater levels of anthocyanins (Fig. 4E). These results suggested that MYB105 promotes the expression of *UFGT*, leading to a shift in the flavonoid metabolic pathway from proanthocyanidins to anthocyanins during the early development stages of the *anr* mutant fruit.

### ANR mutation did not affect sugar accumulation and organic acid content in fruits

**Fig. 5:**
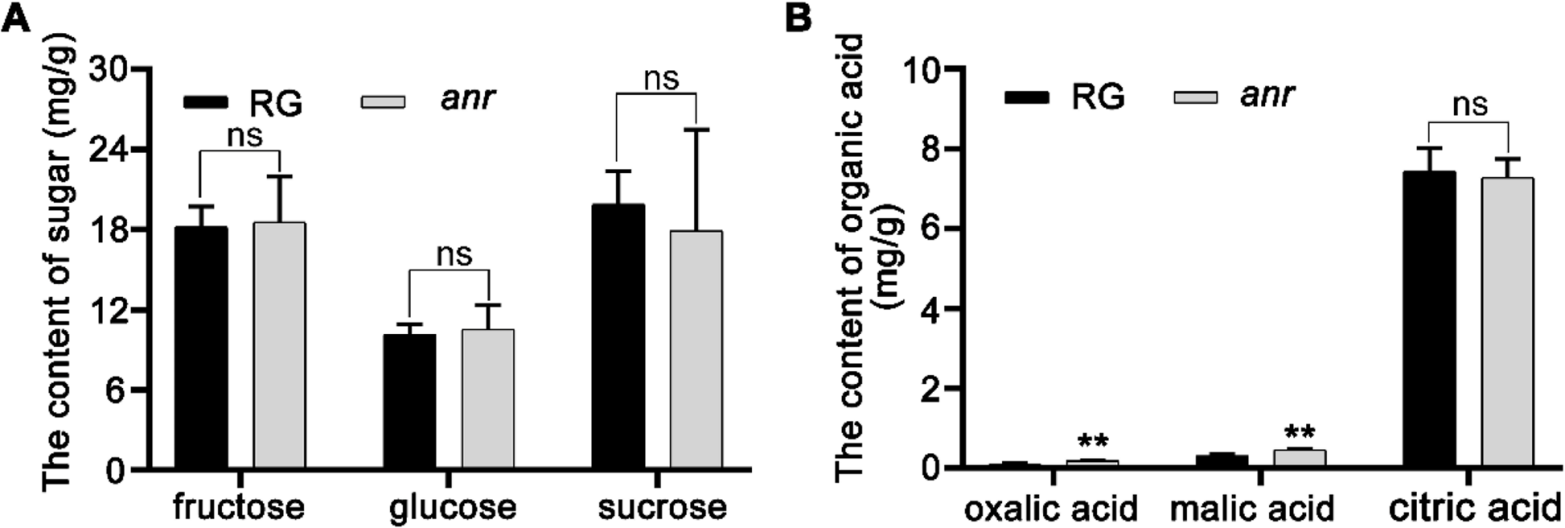
ANR did not affect the sugar and organic acid content of the fruit. **A **The content of sugar in the repining fruits of RG and *anr* mutant. **B** Organic acid content in ripening fruits of RG and *anr* mutant. Data in (**A**) and (**B**) are mean ± SD, *n* = 3. **, *p* < 0.01; ns: no significant. Student's t-test. RG: Ruegen

The quality of fruits is significantly influenced by the sugar content, organic acid levels, and color. In our study, we measured the sugar content in the *anr* mutant fruits and observed no significant differences in the levels of sucrose, fructose, and glucose between *anr* mutant fruits and WT RG fruits (Fig. 5A). Regarding organic acids, although the levels of oxalic acid and malic acid were higher in the *anr* mutant fruits, these acids were present in very low concentrations overall. Moreover, the level of citric acid, which is the predominant organic acid in these fruits, did not significantly differ between the RG and *anr* mutant fruits (Fig. 5B). These results indicated that *ANR* mutation has minimal impact on the sugar and organic acid contents of the fruits, making it a promising target for modifications aimed at altering fruit color without affecting other quality attributes.

### MYB10 only affects fruit phenotype at the ripe stage of the *anr* mutant

**Fig. 6:**
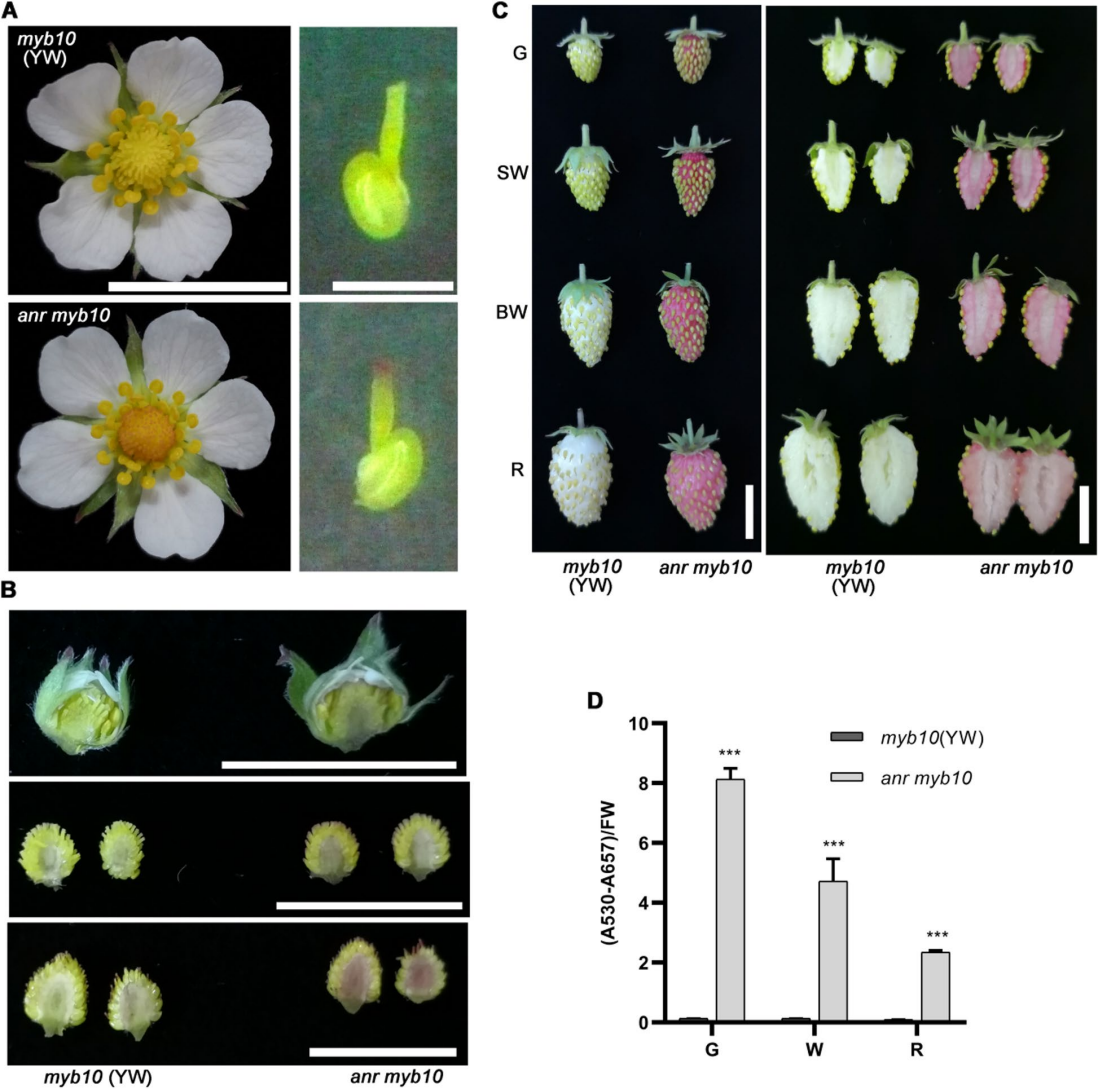
MYB10 does not affect anthocyanin accumulation during early fruit development in *anr* mutant. **A** The phenotype of flower and pistil stigma. **B** The receptacle phenotype in early development stages. **C** The fruits phenotype at different developmental stages. G: green stage, SW: small white stage, BW: Big white stage; R: ripening stage. **D** The content of anthocyanins in fruits at different developmental stages. G: green stage, W: white stage; R: ripening stage. YW: Yellow Wonder, it is a natural mutant of MYB10. FW: Fresh Weigh. Data in (**D**) was mean ± SD, *n* = 3. ***, *p* <0.001, Student’s t-test. Bars in (**A**-**C**) are 1 cm

To further explore the role of MYB10 in *anr* mutants, we crossed the *anr* mutant with Yellow Wonder (YW), a natural mutant lacking functional MYB10, to create the *anr myb10* double mutant. The analysis of this double mutant showed that it exhibited red stigmas in the pistil (Fig. 6A), and no anthocyanin accumulation was observed in the receptacles of the buds and flowers (Fig. 6B, upper and middle panels). However, significant anthocyanin accumulation was noted in the fruit receptacles three days after flowering (Fig. 6B, lower panel), which is consistent with the phenotypes observed in the *anr* (*rg418*) single mutant (Fig. 1A and B).

Further examination of the *anr myb10* double mutant fruits across different developmental stages revealed consistent anthocyanin accumulation during both the green and white stages (Fig. 6C), mirroring the pattern observed in the *anr* single mutant (Fig. 1C). These results indicated that anthocyanin accumulation in the *anr* mutant is independent of MYB10 from the flowering stage through to the white fruit stage. Moreover, the ripened fruits of the *anr myb10* double mutant showed anthocyanin accumulation. However, unlike the RG and *anr* single mutant fruits, which are typically red, the *anr myb10* double mutant fruits exhibited a pink color (Supplemental Fig. S9). Quantitative analysis of the total anthocyanin content revealed a decreasing trend from the green stage to the ripening stage in the *anr myb10* double mutant fruits (Fig. 6D). This contrasts with the fluctuating anthocyanin levels observed in the *anr* single mutant fruits across these stages (Fig. 1D). Overall, these findings suggested that although MYB10 does not affect anthocyanin accumulation from flowering to the white fruit stage in the *anr* mutant, it does affect the color intensity and pattern in mature fruits of the *anr* mutant.

## Discussion

The strawberries currently available in the market are octoploids, formed through crossbreeding and the polyploidization of four diploid wild strawberry species during their evolutionary history. Genome analysis of the octoploid strawberry indicates that the subgenome derived from *F. vesca* is dominant. This subgenome not only retains more genes and shows higher gene expression but also exhibits a bias in homologous chromosome exchanges. Pathway analysis highlighted numerous genes involved in metabolism and disease resistance that are primarily regulated by genes from this subgenome (Edger et al. 2019; Jin et al. 2023). In a study by Labadie *et al.*, metabolome quantitative trait locus mapping was used to identify genes controlling flavonoid metabolites in octoploid strawberry fruits. Their results suggested that the genetic basis for fruit color predominantly is mainly the genes inherited from the *F. vesca*-derived subgenome (Labadie et al. 2022). Therefore, studying the diploid woodland strawberry (*F. vesca*) can advance our understanding and enable the identification of crucial genes that influence fruit color in cultivated octoploid strawberries.

In WT woodland strawberry RG, anthocyanins do not accumulate during the early stages of fruit development; instead, proanthocyanidins are the predominant compounds that accumulate in these fruits. Starting from the green fruit stage, the levels of proanthocyanidins begin to decrease, and the fruits expand, turning white before beginning to accumulate anthocyanins during the turning stage, continuing until the ripening stage. However, even at the ripening stage, only the skin of the fruit turns red, whereas the flesh remains white as it does not accumulate anthocyanins. In our screening of an EMS mutant library, we discovered the *rg418* mutant, which exhibited early-stage anthocyanin accumulation (Fig. 1B). The *rg418* mutant fruits showed significant anthocyanin accumulation in both the skin and flesh from the green stage to the ripening stage (Fig. 1C). Using the MutMap method, we identified *ANR* as the causal gene for *rg418* mutation (Fig. 2 and Supplemental Table S1). Similarly, Fischer *et al*. generated *ANR*-RNAi transgenic plants of octoploid strawberries, which displayed a red stigma during flowering and accumulated anthocyanins starting from the green stage, with mature fruits exhibiting a darker red color (Fischer et al. 2014). These phenotypes are consistent with those observed in the *rg418* mutant, providing strong evidence that *ANR* is the causative gene.

Research has shown that mutations in the anthocyanin transport gene *RAP* can prevent anthocyanin accumulation in both petioles and fruits (Luo et al. 2018). By contrast, the overexpression of *RAP* results in phenotypes similar to those observed in the *anr* mutant (Gao et al. 2020), where *RAP*-overexpressing plants demonstrated significant anthocyanin accumulation in the stigma and receptacle as early as the bud stage (Gao et al. 2020). However, the stigma of the *anr* mutant did not show notable anthocyanin accumulation during the bud stage, akin to the WT RG (Fig. 1B). In addition, significant accumulation of anthocyanins in the receptacle was only observed three days after anthesis (Fig. 1B). This variation in the timing of anthocyanin accumulation might be linked to the use of the strong *35S* promoter, which triggers early and high expression of *RAP*. Given that *RAP* has multiple homologs and is typically expressed during the turning and ripening stages under normal conditions, further research is needed to explore whether the early accumulation of anthocyanins in the *anr* mutant is dependent on *RAP* expression.

The significant accumulation of anthocyanins in the *anr* mutant indicates that while *ANR* is primarily involved in proanthocyanidin synthesis, it also potentially enhances anthocyanin content in fruits. Labadie *et al*. observed a negative correlation between *ANR* expression levels and anthocyanin content in strawberry fruits, identifying an 18-bp motif deletion in the *ANR* promoter region in varieties with darker fruit colors. Utilizing this deletion, they developed a marker for early selection of fruit with darker color in breeding programs (Labadie et al. 2022). In the present study, the *anr* single mutant consistently showed a deeper red color in mature fruits (Fig. 1C), whereas the *anr myb10* double mutant exhibited a pink phenotype in mature fruits (Fig. 6C). Recently, we discovered that a mutation leading to a change from arginine to histidine at position 130 in the flavanone 3-hydroxylase (F3H) protein reduced the enzyme’s conversion capacity, leading to the decreased anthocyanin content and a pink color in mature fruits. However, the *f3h* mutant also displayed a lower sugar content than the WT, suggesting that *F3H* is not an ideal target for developing pink strawberries (Xu et al. 2023). Notably, our findings showed no significant differences in the sugar and organic acid levels between the *anr* single mutant fruits and the WT RG (Fig. 5), making *ANR* a promising target for producing pink-fruited strawberries through biotechnological approaches.

Flavonoid metabolism has three primary branches: anthocyanins, proanthocyanidins, and flavonols (Supplemental Fig. S10). These branches originate from the same metabolic pathway. Thus, changes in one branch can affect the others. We measured flavonoid substances in the petioles and mature fruits of both RG and *anr* mutants. Our findings revealed a significant increase in anthocyanin components and the flavonol quercetin in the *anr* mutant, with a notable decrease in the proanthocyanidin content (Table 1). This shift in the metabolite levels may be due to increased substrate availability for the remaining branches after *ANR* mutation. Three key genes are involved in the final enzymatic reactions for the synthesis of anthocyanins, proanthocyanidins, and flavonols, namely *UFGT*, *ANR*, and *FLS*, respectively (Supplemental Fig. S10). Previous studies have shown that the expression levels of *UFGT* and *FLS* are considerably lower than those of *ANR* during early fruit development (Kang et al. 2013; Hawkins et al. 2017), leading to the accumulation of proanthocyanidins at this stage. In addition, the expression levels of *MYB9*, *MYB11*, and *MYB105* significantly decrease from the green stage to maturity (Xu et al. 2021), indicating their critical role in early fruit development. Using the electronic fluorescent pictograph browser for strawberry gene expression profiles (Hawkins et al. 2017), we found that the expression trends of *MYB9*, *MYB11*, and *MYB105* during early fruit development were similar to those of *ANR* (Supplemental Fig. S11). Our study confirmed that all three MYB transcription factors can bind to the *ANR* promoter and induce its activity. Therefore, our research supports the view that *ANR* expression induced by MYB9, MYB11, and MYB105 during early stages of WT RG fruit development is a key factor in proanthocyanidin accumulation.

The synthesis of proanthocyanidins and anthocyanins shares many enzyme genes and substrates (Supplemental Fig. S10). Across various species, MYB transcription factors that promote proanthocyanidin accumulation can also enhance anthocyanin accumulation (Yue et al. 2023; Jiang et al. 2023; Wang et al. 2022a, b; Tian et al. 2017; Wang et al. 2019). Yue *et al*. found that the transient overexpression of *MYB9/11* could induce anthocyanin accumulation in strawberry fruits, but to a limited extent (Yue et al. 2023). Moreover, yeast one-hybrid experiments revealed that MYB9/11 did not bind to the *UFGT* promoter (Fig. 4A). bHLH transcription factors can form complexes with MYB transcription factors to enhance their binding to enzyme gene promoters in the flavonoid metabolism pathway (Xu et al. 2021; Baudry et al. 2004). Thus, MYB9/11 potentially requires the assistance of bHLH factors to bind to the *UFGT* promoter, but this hypothesis needs further experimental validation. We identified that MYB105 can bind to the *UFGT* promoter and enhance its activity (Fig. 4A and C). Through the transient overexpression of *MYB105* in fruits, we observed a substantial increase in anthocyanin accumulation (Fig. 4D). Moreover, our transcriptome sequencing results showed significantly higher expression levels of both *MYB105* and *UFGT* in the *anr* mutant compared with WT RG (Fig. 3C and D). Furthermore, MYB9, MYB11, and MYB105 have been shown to regulate flavonoid metabolism by binding to the promoters of *CHS2* and *DFR2* (Xu et al. 2021). These existing studies, along with our findings, support the concept that in the *anr* mutant, MYB105 mediates anthocyanin accumulation by increasing the expression of structural genes involved in the anthocyanin synthesis pathway.

We measured anthocyanin content in the *anr* mutant fruits from the green stage to the ripening stage and observed a gradual decrease in anthocyanin content from the green stage to the turning stage, followed by a 2-fold increase during the ripening stage (Fig. 1D). This increase can be attributed to the enhanced expression of *MYB10* during ripening. MYB10 induces the expression of genes related to anthocyanin synthesis, thereby promoting further anthocyanin accumulation in *anr* mutant fruits. To further elucidate MYB10’s role, we examined *anr myb10* double mutant fruits. In these mutants, anthocyanin content gradually decreased from the green stage to the ripening stage without any subsequent increase (Fig. 6D), highlighting MYB10’s critical role during ripening. Compared with the green stage, fruit expansion during the white and turning stages led to a decrease in the anthocyanin content accumulated in the early stages of *anr* mutant fruits. This decrease was more pronounced in the *anr myb10* double mutant (Fig. 6D). The absence of MYB10 impedes anthocyanin accumulation during later stages of fruit development. Thus, as the fruit grows and cell volumes increase, the reduced pigment concentration causes a color transition from red in the early stages to pink at ripening (Fig. 6C and Supplemental Fig. S9).

**Fig. 7:**
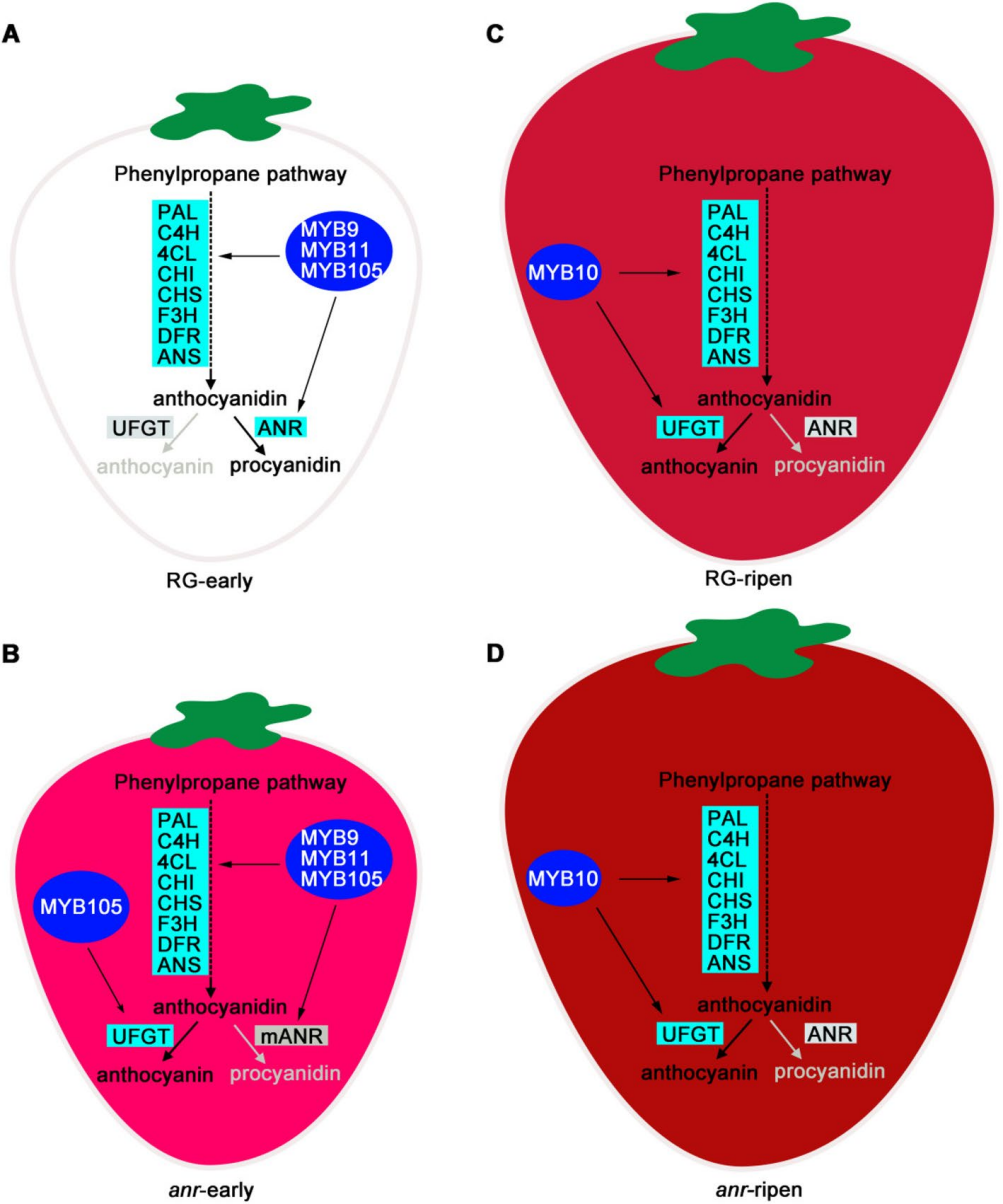
The model of anthocyanin accumulation in RG and *anr* mutant fruits. **A** The pattern of procyanidin accumulation in early development fruits of wild-type RG. **B** The pattern of anthocyanidin accumulation in early development fruits of the *anr* mutant. **C** The pattern of anthocyanidin accumulation in ripe fruits of wild-type RG. **D** The pattern of anthocyanidin accumulation in ripe fruits of the *anr* mutant. During the early stage of fruit development, MYB9/11/105 binds to the promoter of *ANR and* structural genes shared by the flavonoid metabolic pathway, promoting its expression. Meanwhile, the expression level of *UFGT* is low at this stage, leading to a predominant flow of flavonoid metabolism towards procyanidin synthesis. Consequently, a significant amount of procyanidins accumulates in the fruit (**A**). However, in the *anr* mutant, despite the expression level of *ANR* being unaffected, the mutated *ANR* (*mANR*) fails to function as an enzyme protein. This disruption results in the blockage of procyanidin synthesis. Simultaneously, the upregulated expression of MYB105 during this stage enhances the expression of structural genes. This shift in flavonoid metabolism directs the pathway towards anthocyanidin synthesis, leading to the accumulation of abundant anthocyanins in the early development fruits, characterized by a red coloration (**B**). As the fruits progress towards maturity, both RG and *anr* mutant fruits exhibit an increased expression level of *MYB10*. This elevated expression promotes the expression of structural genes, facilitating the accumulation of anthocyanins in the fruits. Notably, RG fruits do not accumulate anthocyanins during the early stage of fruit development. Conversely, *anr* mutant fruits accumulate anthocyanins at this early stage and store them in vesicles until they are superimposed on newly synthesized anthocyanins during the ripening stage. Consequently, the *anr* mutant fruits exhibit a much higher anthocyanin content during the ripening stage compared to wild-type RG fruits, resulting in a deep red coloration (**D**) as opposed to the red coloration observed in wild-type RG fruits (**C**)

Based on the findings of our previous studies and the current results, we have developed a model to illustrate anthocyanin accumulation in both WT RG and *anr* mutant strawberry fruits. In the early stages of fruit development in WT RG, MYB9/11/105 transcription factors activate structural genes within the flavonoid metabolic pathway. These transcription factors also bind to the *ANR* promoter, promoting *ANR* expression. This results in the predominant flow of the flavonoid metabolism pathway into the proanthocyanidin synthesis branch, leading to proanthocyanidin accumulation in the fruits (Fig. 7A). In the *anr* mutant, although *ANR* expression levels do not significantly change (Supplemental Table S4), *ANR* mutation blocks the proanthocyanidin synthesis pathway. The increased expression of MYB105 then triggers a rise in the expression of structural genes, redirecting the flavonoid metabolism pathway toward the anthocyanin branch. This causes an earlier manifestation of red color in *anr* fruits during early development (Fig. 7B). As WT RG fruit progresses to the ripening stage, the enhanced expression of *MYB10* stimulates the expression of enzyme genes involved in the anthocyanin synthesis pathway, promoting anthocyanin accumulation and resulting in red coloration (Fig. 7C). Similarly, in *anr* mutant fruits, MYB10 continues to promote anthocyanin accumulation during later stages of development. The storage of anthocyanins accumulated in the early stages ultimately leads to a deeper red coloration at the ripening stage (Fig. 7D). In addition, *MYB9*/*11*/*105* are primarily expressed in the early stages of fruit development but scarcely expressed during the turning and ripening stages (Xu et al. 2021). By contrast, the gene *MYB10* is mainly expressed in the turning and ripening stages and exhibits minimal expression during early fruit development. This difference in expression trends suggests that the *MYB* genes may not strongly compete with each other. Further investigation is needed to determine whether they bind to the same motifs on the promoters of structural genes.

## Methods

### Plant materials and EMS mutagenesis screening

In this study, we used the seventh generation inbred lines of the woodland strawberry (*F. vesca*) accession Ruegen (RG, red-fruited) as the WT for EMS mutagenesis. Yellow Wonder (YW5AF7, white-fruited) is a naturally occurring loss-of-function mutant of *MYB10*. All strawberry plants were grown in an artificial climate chamber with a photoperiod of 16-h light and 8-h darkness at a temperature of 23°C. For EMS mutagenesis, RG seeds were first soaked for 6 h in water containing 0.2% (v/v) Tween-20 and then transferred to a 0.4% (v/v) EMS solution (Sigma-Aldrich, M0880) for an additional 6 h at room temperature with moderate shaking. After treatment, the seeds were thoroughly rinsed with water until they were odorless. The used EMS solution and the rinse water were treated overnight with 0.5 M NaOH before disposal. The M2 generation was produced by self-fertilizing plants grown from EMS-treated seeds (M1 generation), and mutant plants were screened from the M2 generation plants. For this study, we selected a mutant known as *rg418* because it exhibited a specific anthocyanin accumulation phenotype in the pistil stigma during flower blooming.

### Identification of mutagenic genes using the MutMap method

The *rg418* mutant was crossed with WT RG to obtain F1 hybrid plants. The F1 plants were then self-crossed to produce an F2 population. From the F2 population, 45 plants displaying the mutant phenotype were selected. DNA was extracted from the leaves of each plant by using the CTAB (cetyltrimethylammonium bromide) method (Rogers and Bendich 1985) and mixed in equal amounts to create the mutant pool. DNA was also extracted from RG leaves to serve as the WT pool. Both the WT and mutant pools were sent to Lc-Bio Technologies Co., Ltd (Hangzhou, China) for sequencing. Paired-end 150-bp sequencing was performed using the Illumina HiSeq 2500 platform (Illumina, Inc., San Diego, California, USA). The sequencing data were analyzed using the MutMap pipeline (Sugihara et al. 2022), with the reference genome FvH4 version 4.0.a1 (Edger et al. 2018). According to the MutMap gene mapping method (Abe et al. 2012; Tribhuvan et al. 2018), the candidate SNP index should be 1. Therefore, genes with alterations in their coding regions due to mutation were prioritized as candidate mutagenic genes.

### Plasmid construction

Genomic DNA was extracted from the leaves of WT by using the CTAB method (Rogers and Bendich 1985). A 2000-bp sequence upstream of the ATG codon of *ANR* and *UFGT* was amplified from the genomic DNA to serve as the promoter. These promoter sequences were separately inserted into the *pLacZi* vector to construct *pLacZi-ProANR* and *pLacZi-ProUFGT* for use in yeast one-hybrid assays. Additionally, the promoters of *ANR* and *UFGT* were inserted into the *pGreen0800-LUC* vector to create the reporter vectors *pGreen0800-ProANR::LUC* and *pGreen0800-ProUFGT::LUC* for dual-luciferase assays. Total RNA was extracted from RG fruits at the green stage by using RNA extraction kits (Tiangen, Beijing, China, Cat.DP437), and cDNA was synthesized using reverse transcription kits (Transgen, Beijing, China, Cat.AE311). The full-length CDS of *MYB9*, *MYB11*, *MYB105*, and *bHLH3* were amplified from the cDNA. For the yeast one-hybrid assay, *MYB9*, *MYB11*, and *MYB105* were each inserted into the modified *pB42AD* (*mpB42AD*) vector to construct *mpB42AD-MYB9/11/105*. For transient injection experiments, *MYB9*, *MYB11*, *MYB105*, and *bHLH3* were each inserted into the *pHB-35S::YFP* vector to construct the overexpression vectors *pHB-35S::MYB9/11/105-YFP* and *pHB-35S::bHLH3-YFP*. All plasmids were constructed using the homologous recombination kit (Vazyme Biotech Co., Ltd., Nanjing, China, Cat.C112) and verified through sequencing. The primers used for constructing the plasmids are listed in Supplemental Table S6.

### Transient injection experiments in strawberry fruits

The transient injection assay was conducted following the method reported by Pi et al. (2019), with minor modifications. The vectors *pHB-35S::MYB105-YFP*, *pHB-35S::bHLH3-YFP*, and *pHB-35S::YFP* (negative control) were each transformed into the *Agrobacterium tumefaciens* strain GV3101. A single colony was picked and inoculated into 5 mL of yeast extract peptone (YEP) liquid medium containing rifampicin (50 mg/L) and kanamycin (50 mg/L), and incubated overnight with shaking. The next day, the culture was centrifuged at room temperature; the supernatant was discarded, and the pellet was resuspended in MS liquid medium containing 2% sucrose to reach an OD600 of 0.6. Acetosyringone was added to a final concentration of 200 μM. After activation for 2–3 h at room temperature, the *A. tumefaciens* suspension was injected into white-stage fruits by using a 1-mL syringe. The flesh color phenotype was observed after 7 days. At least six fruits were injected for each vector, and the anthocyanin content was subsequently measured as described in the next section.

### Determination of the anthocyanin, organic acid, and sugar contents

Fruits at various developmental stages were collected according to procedures outlined in previous studies (Xu et al. 2021; Gu et al. 2019). The total anthocyanin content was quantified using the methods described in previous research (Chen et al. 2020; Xu et al. 2021; Li et al. 2020). Mature fruits and petioles from newly expanded leaves of 5-month-old strawberry plants were submitted to Lc-Bio Technologies Co., Ltd., (Hangzhou, China) for pigment component analysis.

The levels of sucrose, glucose, and fructose in the ripening fruits were determined through HPLC (high performance liquid chromatography). For this, 0.3 g of fruit powder was ultrasonicated in 0.8 mL of ultrapure water for 15 min. The mixture was then centrifuged at 12,000 rpm for 10 min at room temperature. The supernatant was filtered through a 0.22-μm membrane, and 20 μL of the filtrate was used for HPLC analysis. The mobile phase consisted of 75% acetonitrile at a flow rate of 0.75 mL/min. A Waters XBridge Amide chromatographic column (3.5 μm) was used, with the column temperature set at 25°C. These analyses were performed in triplicate.

The organic acid content in the ripe fruits was determined through HPLC following the method described by Moing et al. (2001). Three biological replicates were constructed for the assays, with each replicate using at least 6 fruits and 6 petioles from three plants.

Fruits harvested 3–5 days after flowering were obtained from 6-month-old RG and *anr* mutant plants. The receptacles, with the carpels removed, were used for total RNA extraction. Magnetic beads with Oligo (dT) were employed to enrich mRNA, which was then fragmented into shorter fragments. Single-stranded cDNA was synthesized using random hexamers, followed by the synthesis of double-stranded cDNA. The double-stranded cDNA was purified, end-repaired, and ligated with sequencing adapters. Fragment size selection was performed using AMPure XP beads, and the final cDNA library was enriched through PCR. Paired-end reads were generated by sequencing the libraries with DNBSEQ-T7 at Beijing Biomics Tech Co., Ltd. (Beijing, China).

Raw reads from each library were individually filtered following the previously described protocol (Xu et al. 2018). The resulting clean reads were mapped to version 6.0 of the woodland strawberry reference genome (Zhou et al. 2023), and gene expression levels were estimated using FPKM. DEGs between RG and *anr* mutants were determined using DEGseq (Wang et al. 2010), and adjustments were made for multiple testing with the Benjamini-Hochberg false discovery rate (FDR < 0.05). Significant DEGs were selected on the basis of a fold change of ≥2 and an adjusted p value of ≤0.05. Pathways involving DEGs were annotated using the KEGG database, with an E-value threshold of 10^−5^. The raw datasets have been deposited in the BioProject of the National Center for Biotechnology Information (NCBI) under accession number PRJNA998615.

### Real-time fluorescence quantitative PCR assays

Total RNA was extracted from fruits by using the RNA Plant Plus Reagent kit (Tiangen, Beijing, China, Cat. DP437) and reverse transcribed into cDNA by using the One-Step gDNA Removal and cDNA Synthesis SuperMix kit (Transgen, Beijing, China, Cat. AE311) following the manufacturer’s instructions. The resulting cDNA was diluted five times, and 1 μL was used for real-time qPCR experiments, as previously reported (Xu et al. 2021). At least six fruits from three plants were used for each replicate, with three independent biological replicates and three technical repetitions for each. Gene expression trends were derived from the three independent biological replicates. The primer sequences reported in a previous study (Xu et al. 2023) were used in this study.

### Yeast one-hybrid assays

The effector plasmids *mpB42AD-MYB9/11/105* and the reporter plasmids *pLacZi-ProANR* or *pLacZi-ProUFGT* were co-transformed into EGY48 yeast cells and grown at 30°C for 3 days. Transformed colonies were selected on SD-Trp-His-Ura medium supplemented with X-gal, as described previously (Li et al. 2020).

### Dual-luciferase reporter assays

The reporter strain GV3101 harboring *pGreen0800-ProUFGT::LUC* was mixed with each effector strain harboring *pHB-35S::YFP* (negative control*), pHB-35S::MYB105-YFP*, or *pHB-35S::bHLH3-YFP* plasmids individually as well as with combinations of these plasmids as indicated. Each combination was infiltrated into tobacco (*Nicotiana benthamiana*) leaves. After incubation in the dark for 36–40 h, the leaves were harvested and ground into powder in liquid nitrogen for LUC activity determination. The assay was conducted using the Dual Luciferase Reporter Gene Assay Kit (Cat.11402ES60, Yeasen Biotechnology, Shanghai, China) following the manufacturer’s instructions. The LUC activity of MYB9/11/105 on the *ANR* promoter was measured as described above. All assays were performed with at least three biological replicates.

### Alignment analyses

The *ANR* sequences from RG and the *rg418* mutant were obtained through PCR. The CDS and protein sequences of *ANR* were compared using Muscle software (Edgar, 2022), and the results were displayed using BioEdit software.

### Statistical analyses

All statistical analyses were conducted using GraphPad Prism 8 software. Student's t test was used for pairwise comparisons (*, *P* < 0.05; **, *P* < 0.01; ***, *P* < 0.001). Tukey’s test (*P* < 0.05) and one-way analysis of variance were used for multiple comparisons.

## Supplementary Information

Supplementary Material 1.

Supplementary Material 2

Supplementary Material 3

Supplementary Material 4

Supplementary Material 5

Supplementary Material 6

Supplementary Material 7

## Data Availability

The data will be available from the corresponding author upon reasonable request.

## Abbreviations

EMS: Ethyl methanesulfonate
WT: Wild-type
RG: Ruegen
BSA-Seq: Bulked sergeant analysis sequencing
ANR: Anthocyanidin reductase
UFGT: UDP-glucose flavonoid 3-O-glucosyltransferase
GDS: Genomic database for strawberries
CHS: Chalcone synthase
CHI: Chalcone isomerase
F3H: Flavanone-3-hydroxylase
DFR: Dihydroflavonol-4-reductase
ANS: Anthocyanidin synthase
MBW: MYB-bHLH-WD40
CDS: Coding sequence
RAP: Reduced anthocyanins in petioles
SNP: Single nucleotide polymorphism
PAL: Phenylalanine ammonia lyase
RT-qPCR: Reverse transcription-quantitative polymerase chain reaction
FPKM: Fragments per kilobase per million
DEGs: Differentially expressed genes
LUC: Luciferase
YW: Yellow wonder
CTAB: Cetyltrimethylammonium bromide
HPLC: High performance liquid chromatography
