# Supplementary material for: Loss-of-function mutation in anthocyanidin reductase activates the anthocyanin synthesis pathway in strawberry
Source: Mol Hortic. 2024 Sep 14;4:33. doi: 10.1186/s43897-024-00106-2 (PMC11401314; doi:10.1186/s43897-024-00106-2)
Supplement: Supplementary file 1 — Supplementary Material 1. [file 43897_2024_106_MOESM1_ESM.docx]

**Supplementary Informations**


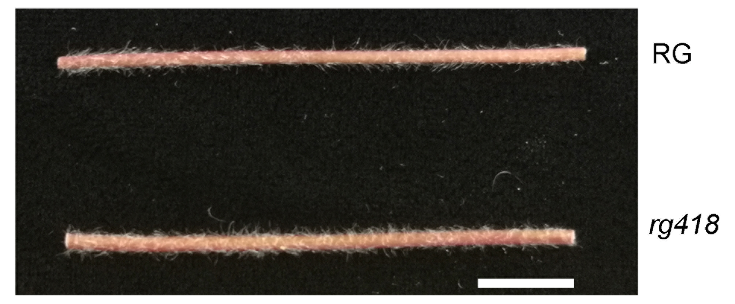


Supplemental Fig.S1. The anthocyanin phenotype in petiole of RG and *rg418* mutant. Bar is 1 cm.


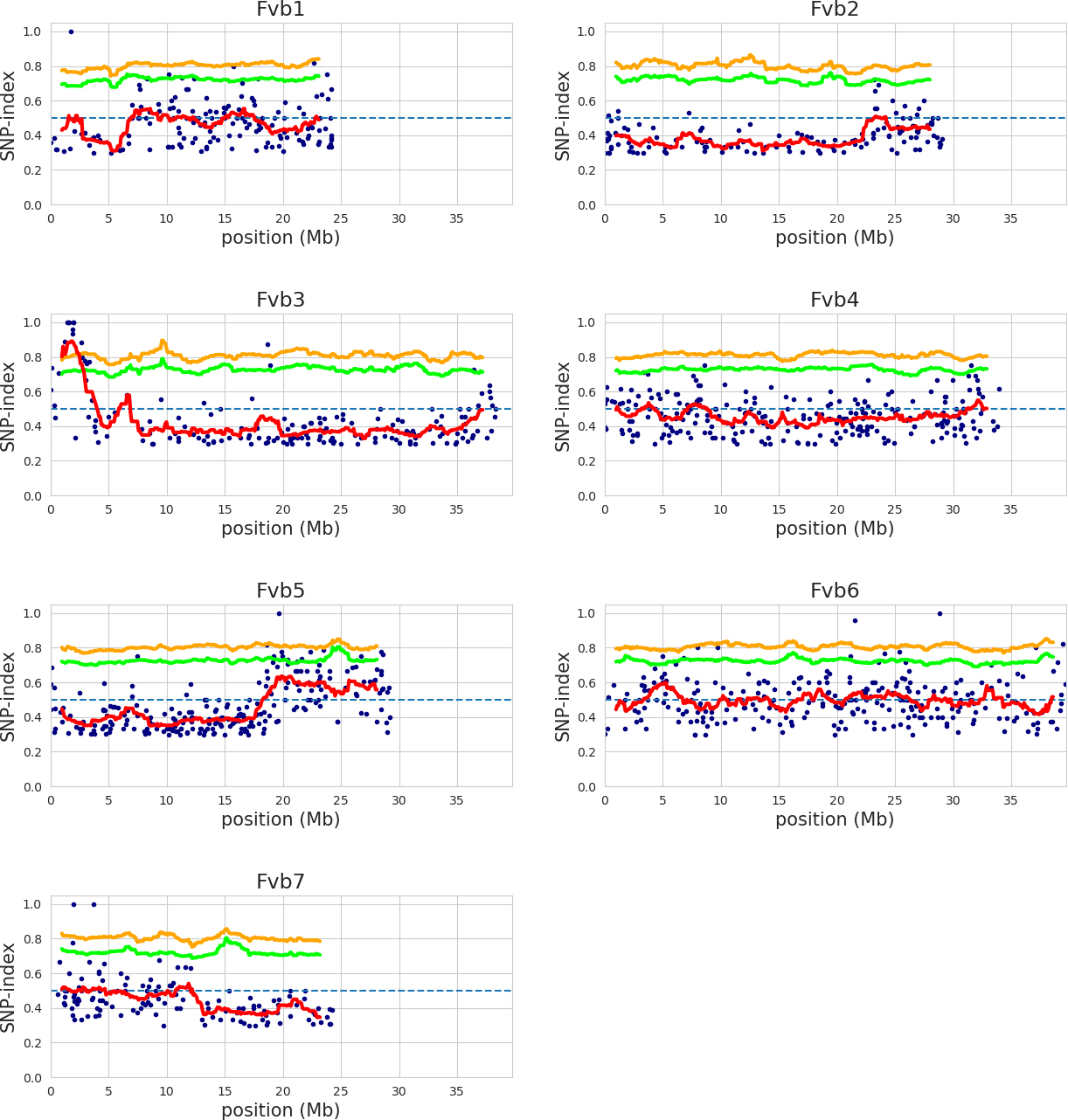


Supplemental Fig. S2. The mapping of mutant gene of *rg418* mutant by Mutmap pipeline. The vertical axis indicates the value of SNP-index; the horizontal axis represents the position of the SNP on the chromosome. The red line represents the mean of SNP-index. The orange line and green line represent the mean p-value of SNP was 0.01 and 0.05, respectively.


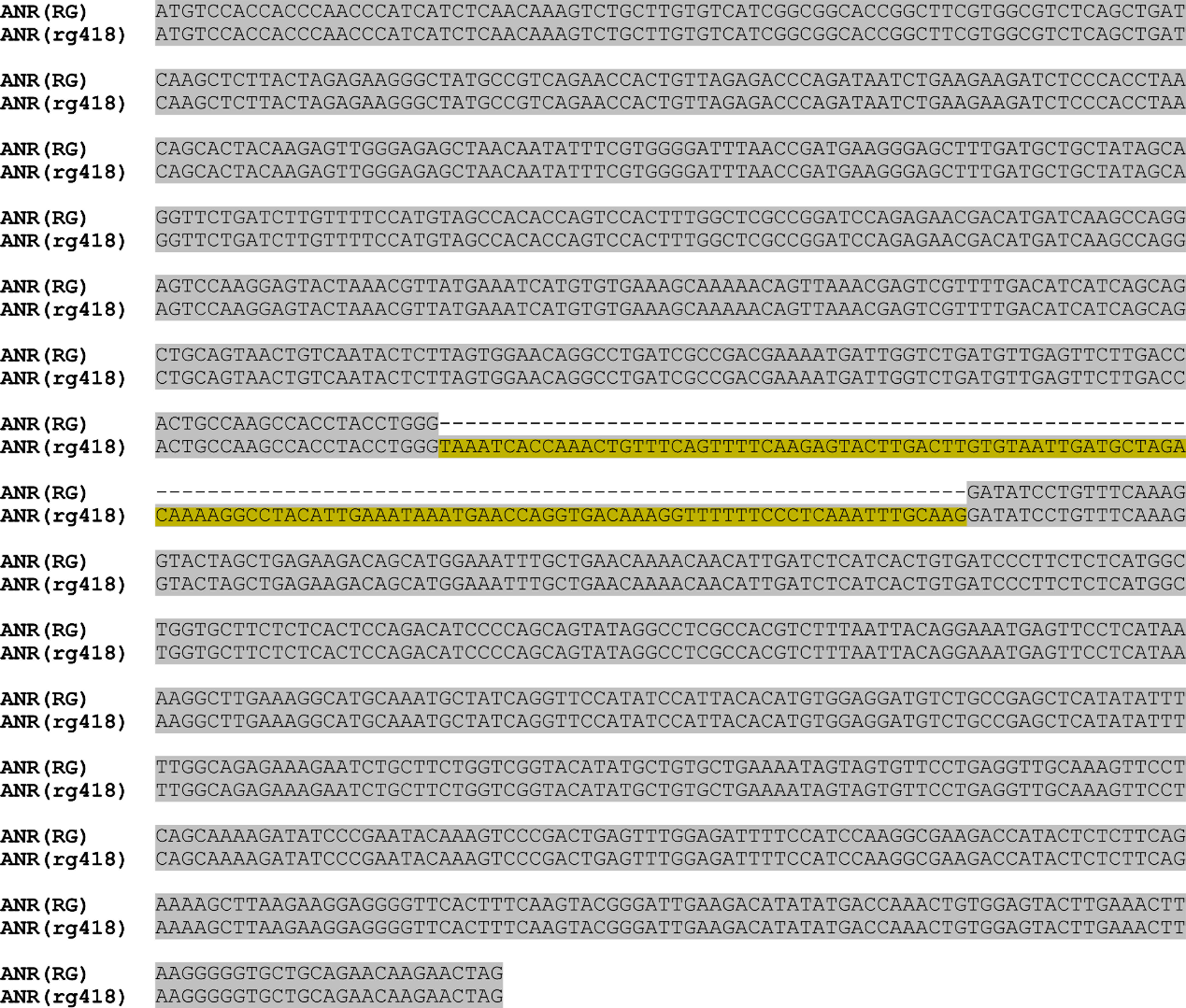


Supplemental Fig.S3. Alignment of ANR full-length CDS sequences from RG and *rg418* mutant. Sequences in yellow represent intron sequences that were retained in *rg418* mutant.


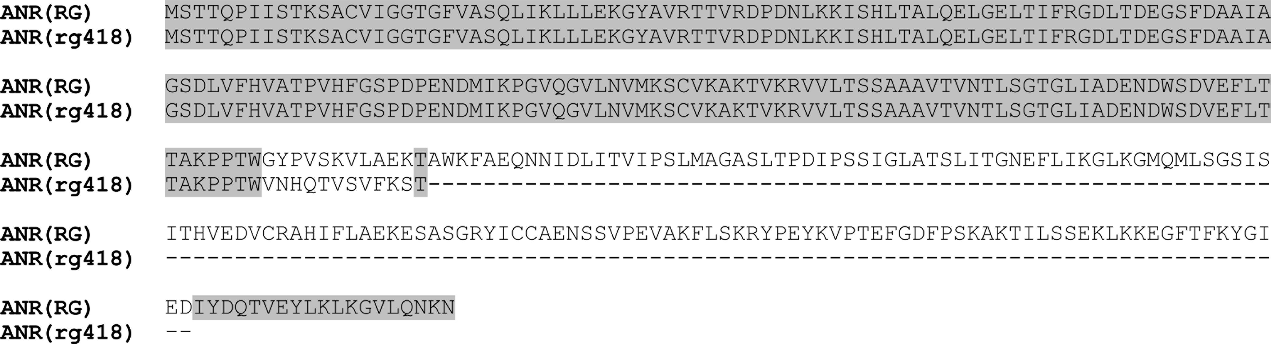


Supplemental Fig.S4. Alignment of ANR protein sequences from RG and *rg418* mutant. The retention of the intron sequence in the *rg418* mutant allows the protein of ANR to undergo a frame shift mutation, leading to its premature termination.


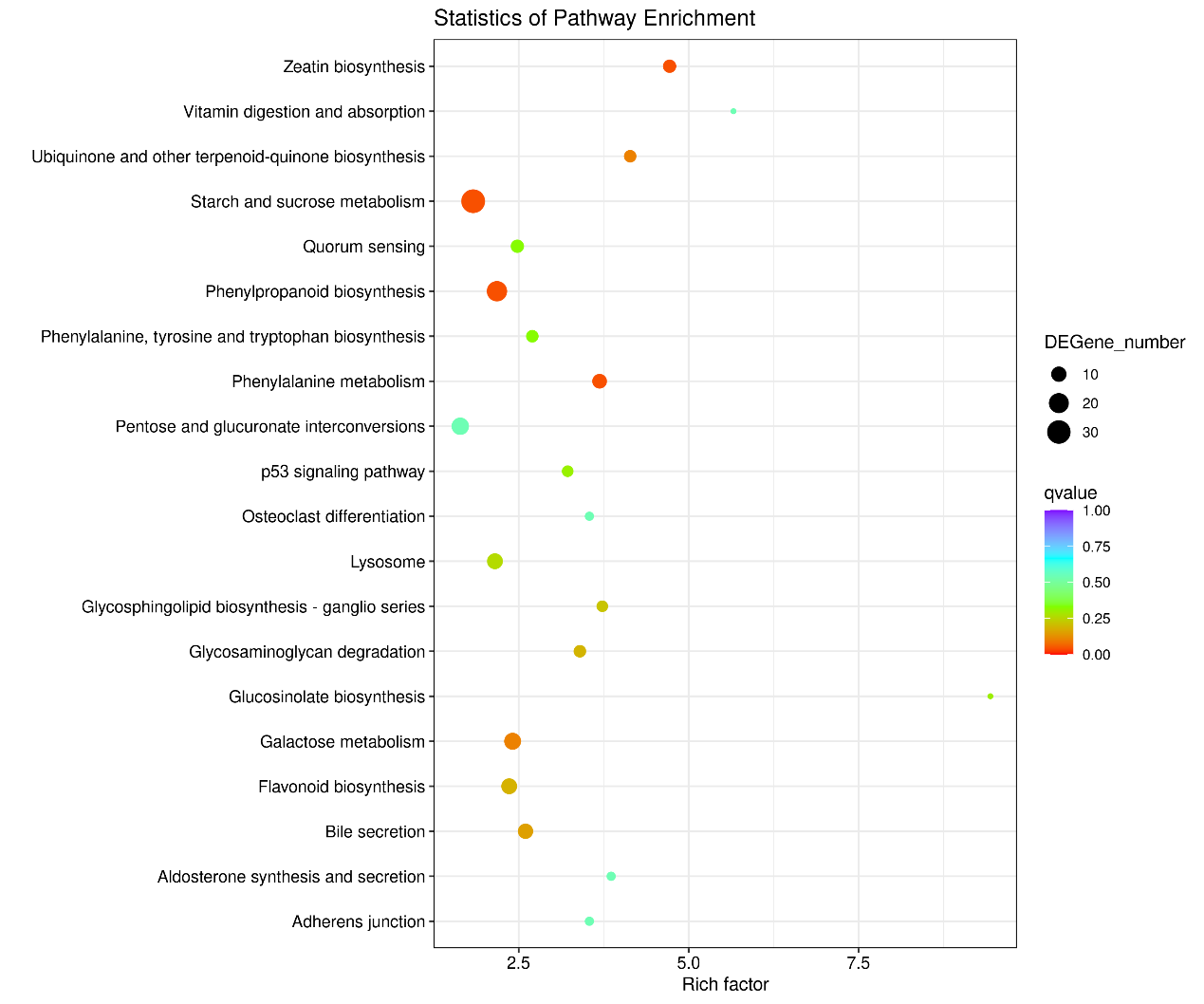


Supplemental Fig.S5. Pathway enrichment analysis of differential genes in *anr* mutant and RG early developing fruits.


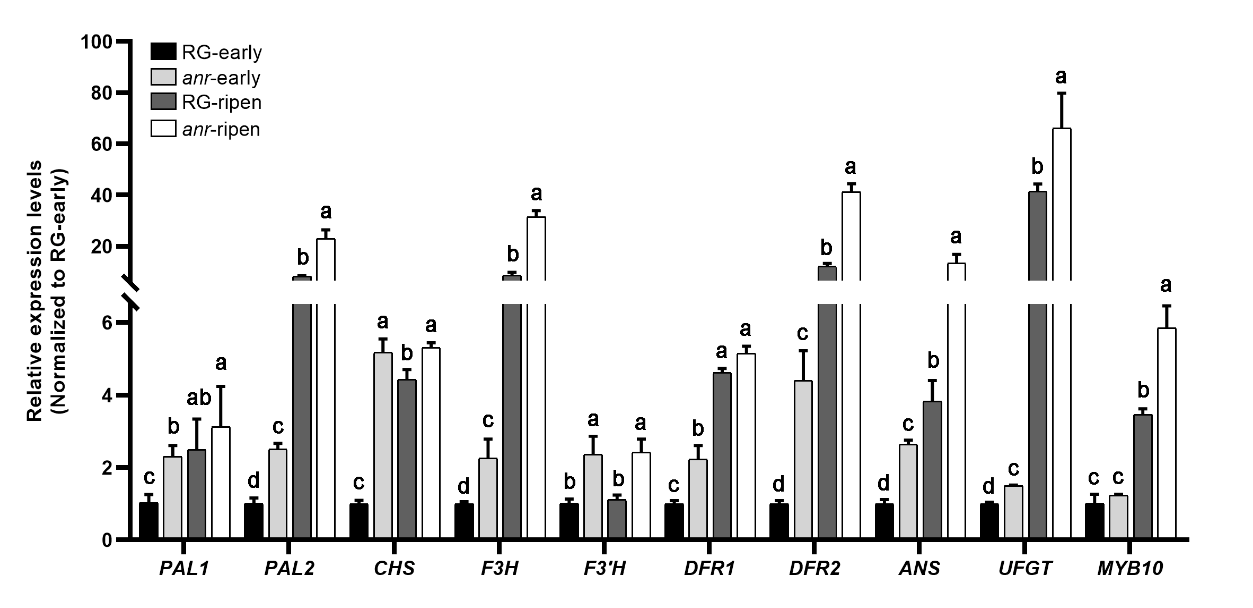


Supplemental Fig.S6. The relative expression levels of anthocyanin synthesis-related enzyme genes in RG and *anr* mutant were examined by RT-qPCR.The gene expression levels of RG early development fruits were normalized to 1. early: early development fruits; ripen: ripen stage fruits. Different letters indicate significant differences between different groups (one-way ANOVA, Tukey test, *p*< 0.05).


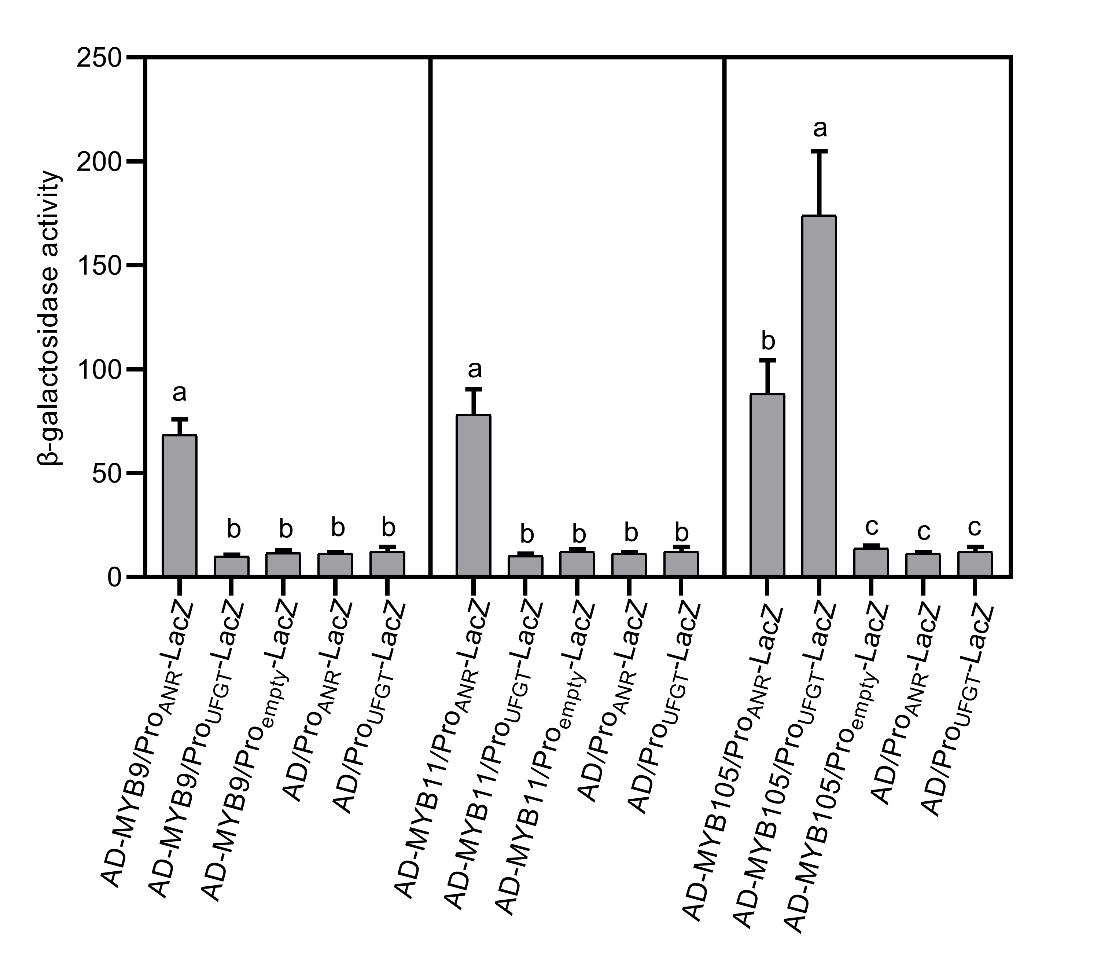


Supplemental Fig.S7. Quantification of β-galactosidase activity for the yeast one-hybrid assay in Figure 4A. Different letters indicate significant differences between different groups (one-way ANOVA, Tukey test, *p*< 0.05).


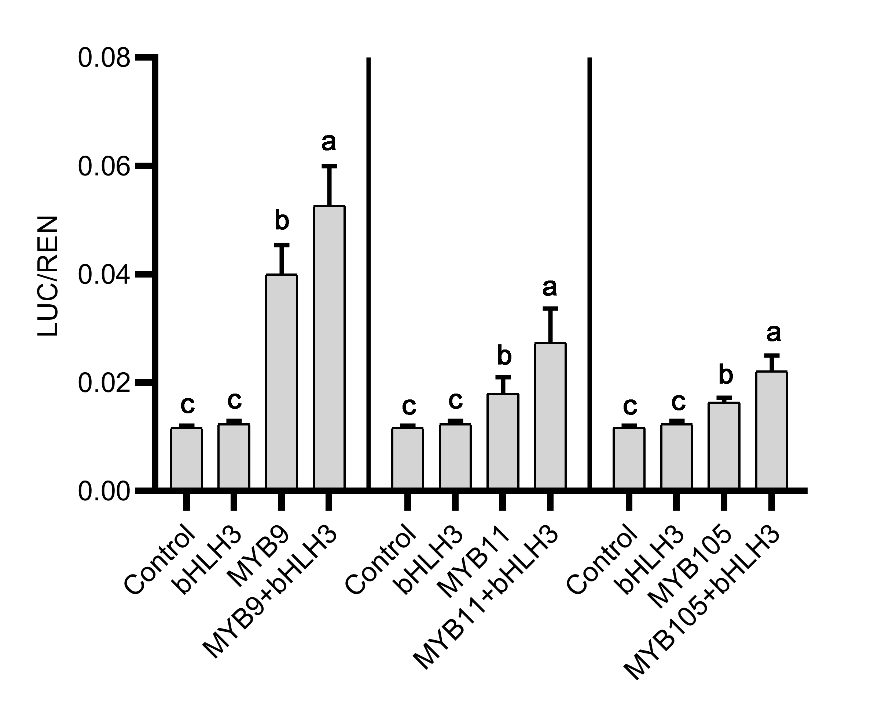


Supplemental Fig.S8. MYB9/11/105 activates the promoter of *ANR*. The vertical axis indicates the ratio of firefly luciferase enzyme activity to renilla luciferase enzyme activity. REN: reilla luciferase; LUC: firefly luciferase. 35S-driven REN was used as an internal control. Different letters indicate significant differences between different groups (one-way ANOVA, Tukey test, *p*< 0.05).


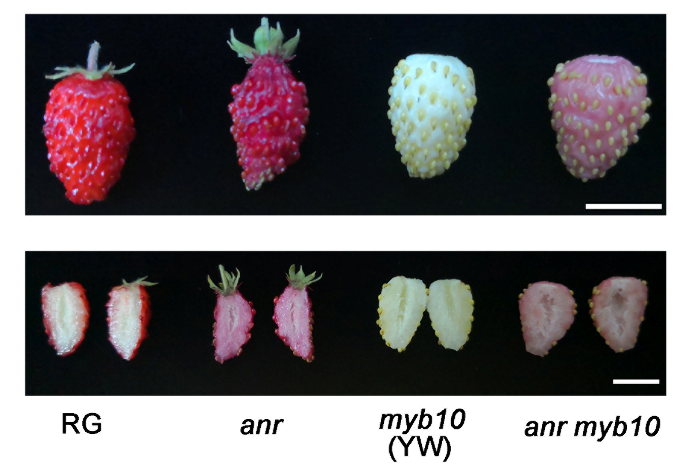


Supplemental Fig.S9. The phenotypes of fruits at maturity stage among different genotypes. YW: Yellow Wonder, it is a natural mutant of MYB10. Bars are 1 cm.


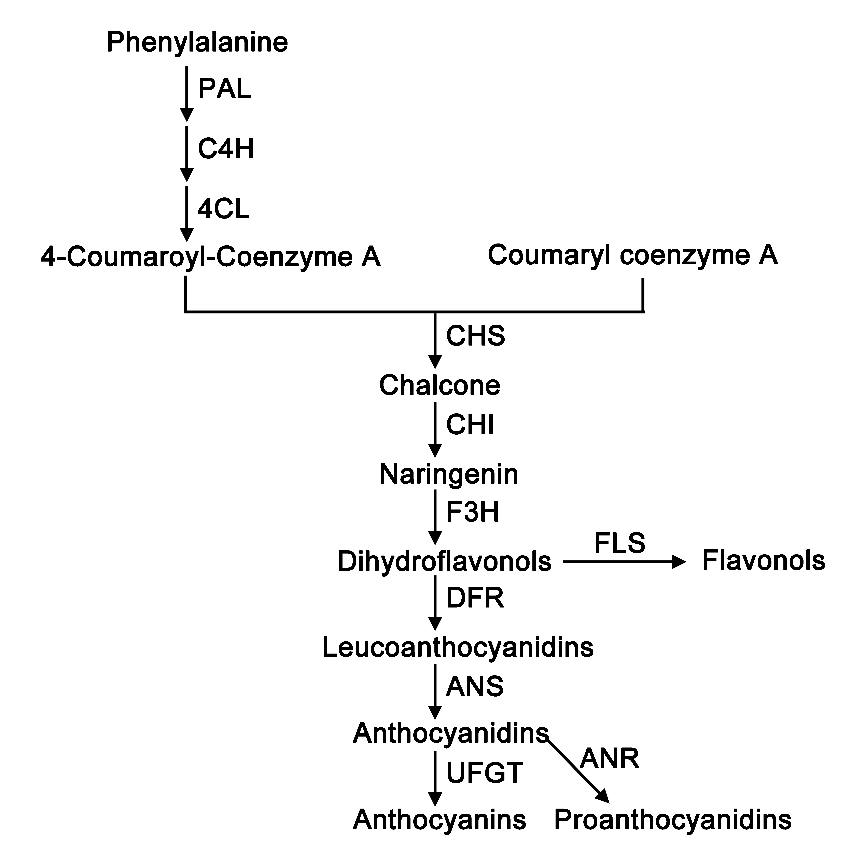


Supplemental Fig.S10. Simplified flavonoid biosynthetic pathway. PAL, phenylalanine ammonia-lyase; C4H, cinnamic acid-4-hydroxylase; 4CL, 4-coumarate-CoA ligase; CHS, chalcone synthase; CHI, chalcone isomerase; F3H, flavanone-3-hydroxylase; DFR, dihydroflavonol-4-reductase; ANS, anthocyanidin synthase; UFGT, UDP-glucose flavonoid-3-O-glucosyltransferase; ANR, anthocyanidin reductase; FLS, flavonol synthase.


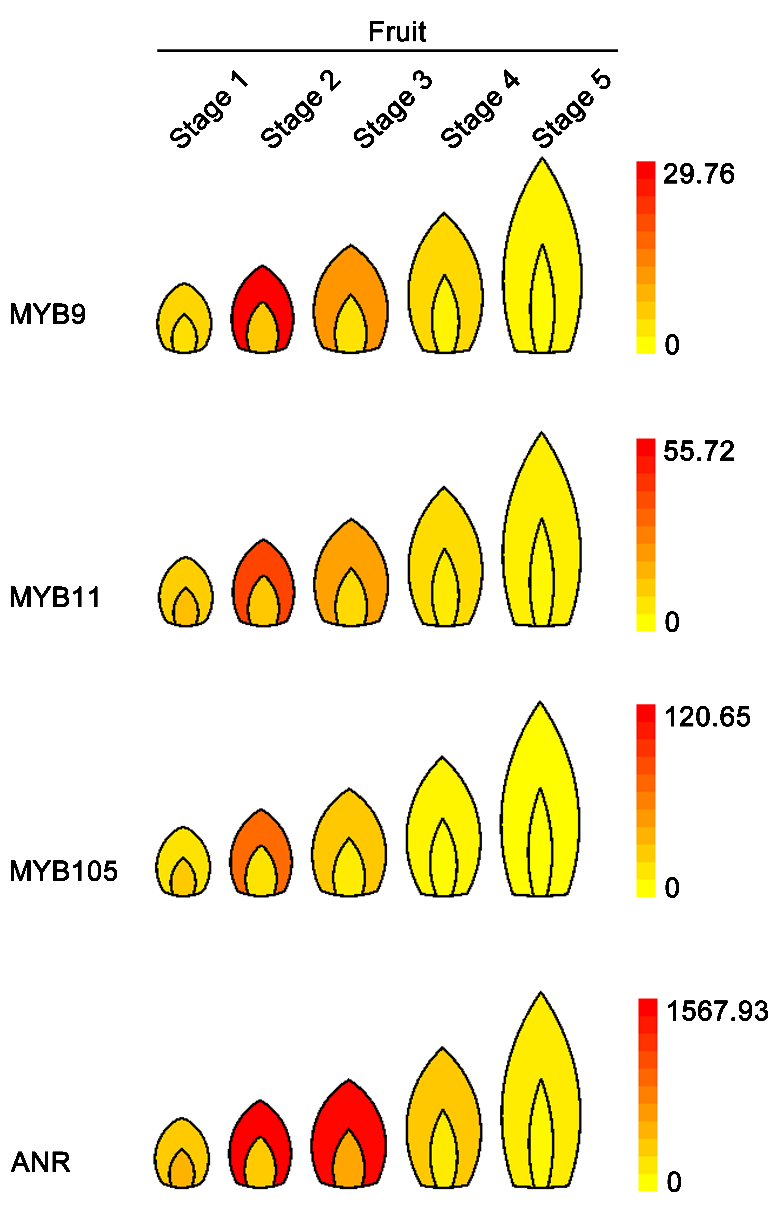


Supplemental Fig.S11. The expression profiles of MYB9/11/105 and ANR in early-stage development of fruits. The data are from the eFP (electronic fluorescent pictograph) browser for strawberry gene expression profiles (Hawkins et al., 2017). Strawberry fruits are formed by the development and expansion of the receptacle. The outer of receptacle was cortex; and the inner of receptacle was pith.
